# Dosimetry of heavy ion exposure to human cells using nanoscopic imaging of double strand break repair protein clusters

**DOI:** 10.1038/s41598-022-05413-6

**Published:** 2022-01-25

**Authors:** Judith Reindl, P. Kundrat, S. Girst, M. Sammer, B. Schwarz, G. Dollinger

**Affiliations:** 1 Institute for Applied Physics and Measurement Technology, Universität Der Bundeswehr München, Neubiberg, Germany; 2 Department of Radiation Dosimetry, Nuclear Physics Institute CAS, Prague, Czech Republic

**Keywords:** DNA damage and repair, Super-resolution microscopy

## Abstract

The human body is constantly exposed to ionizing radiation of different qualities. Especially the exposure to high-LET (linear energy transfer) particles increases due to new tumor therapy methods using e.g. carbon ions. Furthermore, upon radiation accidents, a mixture of radiation of different quality is adding up to human radiation exposure. Finally, long-term space missions such as the mission to mars pose great challenges to the dose assessment an astronaut was exposed to. Currently, DSB counting using γH2AX foci is used as an exact dosimetric measure for individuals. Due to the size of the γH2AX IRIF of ~ 0.6 µm, it is only possible to count DSB when they are separated by this distance. For high-LET particle exposure, the distance of the DSB is too small to be separated and the dose will be underestimated. In this study, we developed a method where it is possible to count DSB which are separated by a distance of ~ 140 nm. We counted the number of ionizing radiation-induced pDNA-PKcs (DNA-PKcs phosphorylated at T2609) foci (size = 140 nm ± 20 nm) in human HeLa cells using STED super-resolution microscopy that has an intrinsic resolution of 100 nm. Irradiation was performed at the ion microprobe SNAKE using high-LET 20 MeV lithium (LET = 116 keV/µm) and 27 MeV carbon ions (LET = 500 keV/µm). pDNA-PKcs foci label all DSB as proven by counterstaining with 53BP1 after low-LET γ-irradiation where separation of individual DSB is in most cases larger than the 53BP1 gross size of about 0.6 µm. Lithium ions produce (1.5 ± 0.1) IRIF/µm track length, for carbon ions (2.2 ± 0.2) IRIF/µm are counted. These values are enhanced by a factor of 2–3 compared to conventional foci counting of high-LET tracks. Comparison of the measurements to PARTRAC simulation data proof the consistency of results. We used these data to develop a measure for dosimetry of high-LET or mixed particle radiation exposure directly in the biological sample. We show that proper dosimetry for radiation up to a LET of 240 keV/µm is possible.

## Introduction

The usage of nuclear technologies in the fields of energy production, research, and human health has constantly been increasing in the last decades. Especially technologies using high- or mixed linear energy transfer (LET) particle bunches, such as novel tumor therapy methods using heavy ions^1^, are of increasing importance. Despite strict and expanded safety regulations, unplanned human exposure might occur due to equipment failure or other accidents. Furthermore, unexpected human exposure to radiation can occur due to attacks with atomic bombs. The prompt and accurate identification and quantification of the dose and radiation quality, an individual was exposed to, is essential for the initial medical assessment and treatment decision^2,3^. Especially in cases where the exposed human did not wear a dosimeter, or when there are discrepancies between the technical measurements using dosimeters and occurring radiation injuries, additional biological dosimetry methods are necessary^4^. Furthermore, planned manned missions to outer space, such as the Mars mission, pose a great challenge for the dose estimation of the astronauts, which is necessary to be able to assess the possible health effects to the individual and possibly take countermeasures^5,6^.

Therefore, there is a need for fast, simple, and scalable biological dose assessment methods for triage, which was found to be the counting of double-strand breaks (DSB) via protein modifications or accumulation upon radiation. These accumulations can be labelled, counted and correlated to the applied dose in single cells. Compared to the other biological dosimetry methods based on cytogenetic assays, the foci assay does not have to rely on the fact that the cells go into cell division once more^3^, but directly relies on the damage recognition and the start of the repair. This is of special importance after high-LET particle exposure as this significantly alters cell division. The conventional foci assay for dose assessment, in this case, is the γH2AX foci assay for a cell sample collected from the exposed human^7,8^. The histone H2AX is phosphorylated upon DSB induction to form γH2AX^9,10^, and serves as a signal for DSB recognition^11^. The phosphorylation takes place in an Mbp (megabase pair) range around the damage. It can be detected using antibody labeling and fluorescent microscopy, where the phosphorylated region appears in so-called foci or ionizing radiation-induced foci (IRIF)^12^. For low-LET radiation (commonly considered as LET < 10 keV/µm), there is a direct correlation between the number of γH2AX foci and the dose, as the ionization density is low for this kind of radiation and the DSB are well separated^13–15^. The size of a single γH2AX focus is ~ 0.5–0.6 µm full width at half maximum (FWHM), which means that DSB can be discriminated and counted only as long as they are separated by this distance^16^. When the DSB distance is smaller, which means that the DSB density gets larger, the number of γH2AX foci is smaller than the number of DSB^17^, and therefore the absorbed dose will be underestimated. A further IRIF counting assay for biodosimetry consists in using damage recognition proteins, which form clusters around the DSB. The conventionally used one is 53BP1, which accumulates in Mbp large regions around the damage downstream of H2AX phosphorylation^17^. It is known to accumulate to every DSB a few minutes after irradiation^18^, and can therefore also be used as a biodosimetric marker^19–22^. Unfortunately, 53BP1 forms IRIF of the same size as γH2AX^16^ and is therefore bound to the same limitations. Both methods are therefore only reliable for low LET radiation.

High LET particles (LET > 10 keV/µm), which are present e.g. in galactic cosmic rays, in the radiotherapy with carbon ions, in the fast neutron irradiation from atomic bombs, or in the α-radiation from natural radon exposures, induce more than one DSB along with the ion traversal through a cell. The density of induced DSB in the tracks increases with increasing LET, which means that more than one DSB can be formed per micrometer track length^22,23^. This is the region where the γH2AX and 53BP1 assays fail, and a new, reliable and small label for DSB must be found.

A prominent candidate is the catalytic subunit of the DNA protein kinase (DNA-PK) phosphorylated at the phosphorylation site T2609. DNA-PK is formed by the Ku heterodimer and the catalytic subunit (DNA-PKcs) and is activated in response to DSB induction via ionizing radiation^24^. Upon DSB induction, the Ku heterodimer, consisting of Ku70 and Ku80, binds to the DNA ends, thus functioning as a DNA-binder and stimulating DNA-PKcs kinase activity^25^. On the large DNA-PKcs protein there are several phosphorylation sites, which are phosphorylated by different kinases such as ATR, ATM, or DNA-PKcs itself, depending on the damaging agent as well as the setup, in-vitro or in-vivo^26^. These phosphorylation sites are grouped in clusters. Phosphorylation of DNA-PKcs at the ATM-dependent phosphorylation site T2609, as part of the ABCDE group, regulates its activity upon irradiation and gives the opportunity to specifically label the region of a DSB. The formed phosphorylated DNA-PKcs (pDNA-PKcs) IRIF can be visualized using antibody labeling and microscopy, which was proven in various studies using different kinds of antibodies, imaging and immunocytochemistry techniques^24,27–29^. Due to its responsibility directly at the damage location, pDNA-PKcs is meant to form smaller foci than 53BP1, which may be used for counting damage and thus for biodosimetry at higher DSB densities^30^.

There are microscopy methods to investigate the protein clustering to a single DSB. Transmission electron microscopy can detect a single DSB by imaging the gold-labeled Ku70/80 heterodimer^31^. This method has the disadvantage that expensive sample processing is necessary, which makes it unalluring for biodosimetric use. Optical super-resolution methods can be used either by imaging protein clusters using immunofluorescence^32–34^ or in combination with pre extraction^35^. The possibility to image whole and intact cell nuclei makes these methods ideal candidates for a visual method of biodosimetry. Super-resolution STED microscopy was recently used to investigate the spatial clustering and temporal accumulation of various DSB repair proteins and their role throughout DSB repair in different studies^16,36,37^. The achieved resolution of 100 nm opens the possibility to get a deeper insight into the orchestration of proteins involved in DSB recognition and repair, as accumulations and structures of this small size can be detected and investigated in the whole cell for the first time.

The simulation of damage induction by high-LET particles by the use of Monte-Carlo methods, such as PARTRAC^38^ or the local effect model (LEM)^39^ is an important tool in radiobiological research. The simulation, on the one hand, helps to understand the basic processes but on the other hand, can serve as a database for therapy planning^39^ and also for quantification of damage induction^40^. The simulation tool PARTRAC provides the type and position of each damage induced by the simulated ion, which makes it essentially a DSB based dosimetry tool.

In this study, we investigate the possibility to use pDNA-PKcs as an alternative for γH2AX and 53BP1 in the biodosimetry of high-LET particle exposure with a focus counting assay using super-resolution STED microscopy. The main advantage here is that the size of pDNA-PKcs IRIF is much smaller than that of 53BP1 and γH2AX.

The reliability of this new assay is proven by comparing the numbers of induced foci for pDNA-PKcs and 53BP1 using different doses of low-LET γ-radiation. For high-LET particles the linear IRIF density (LID), namely the number of IRIF per µm-track length is determined. To be able to perform a dosimetric analysis from this data, it is necessary to be able to use the LID to define the particle LET. Ideally, this can be done using a measured database, where the linear IRIF density is determined depending on LET and ion energy for different ion types. Establishing such a database is a complex and effortful project. We show the first proof-of-principle experiment, which proves that the idea of high-LET particle biodosimetry using IRIF counting works at least in human HeLa cells and then start to build such a database. Although the size of DNA-PKcs IRIF is much smaller than that of γH2AX and 53BP1 IRIF it still limits the resolution to differentiation DSB that are induced within this distance forming DSB clusters. We used the data of clusters of DSB with the same size as the measured IRIFs from Monte Carlo simulations with PARTRAC^38^ as a direct comparison and calibration. This software has recently been used to correlate induced DSB, as provided by the simulation, with γH2AX foci after neutron and photon irradiation^41^.

For dosimetry, the particle LET is determined by comparing the measured LID with the simulation which defines the relation between linear cluster density and LET. This LET value together with particle fluence, which is also read from the images, is used to determine the applied dose. This new method of IRIF counting and dosimetry is tested and applied on high-LET particle-induced damage irradiations in human HeLa cells. This consequently opens the unique opportunity to do dosimetry on high-LET particle tracks, which was also performed here.

## Results

### pDNA-PKcs as a dosimetry marker for low-LET radiation

**Figure 1:**
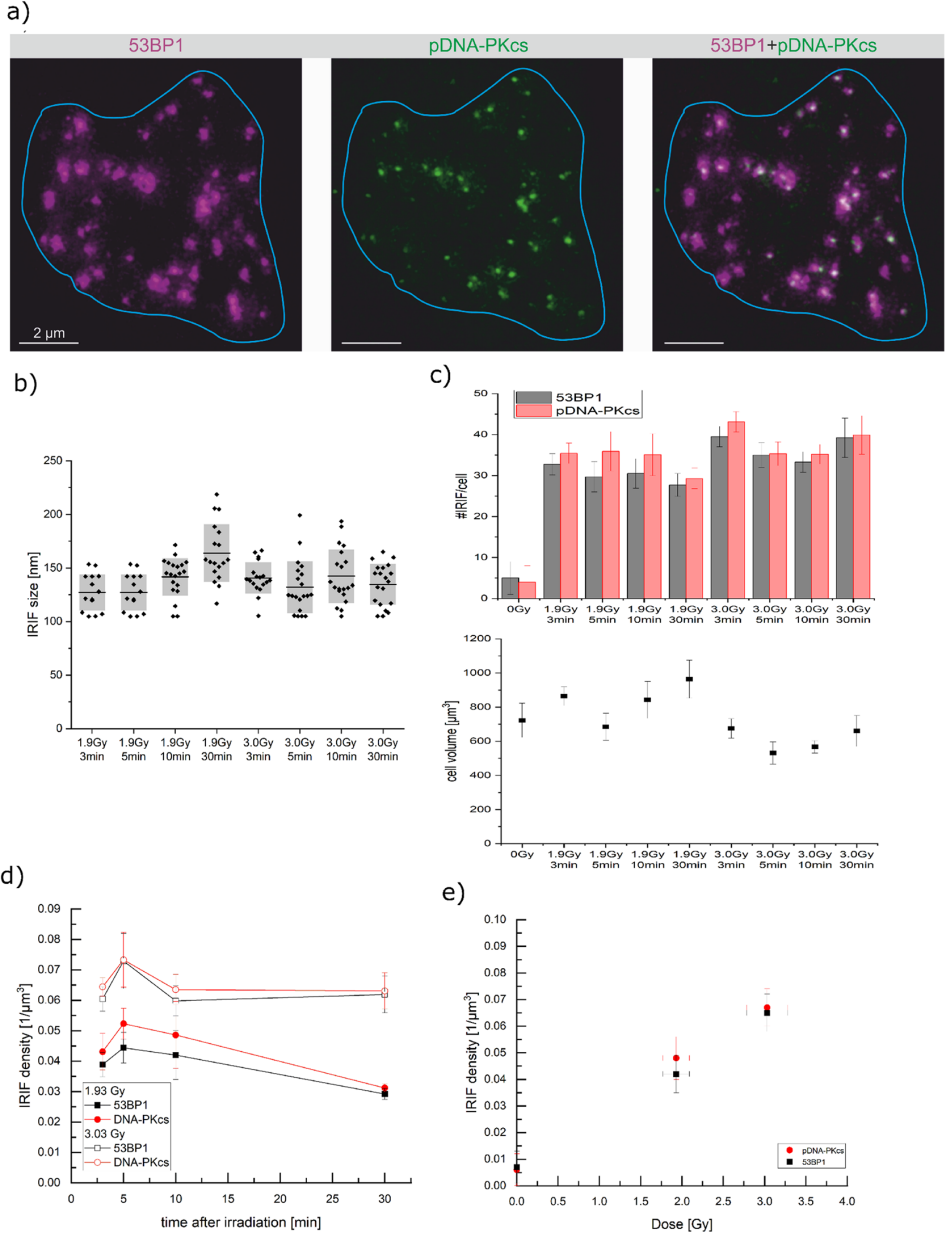
(**a**) 2D section of a HeLa cell nucleus irradiated with 3.0 Gy γ-radiation. The blue line represents the cell nucleus. For each 53BP1 IRIF (magenta), at least one pDNA-PKcs IRIF (green) is located inside the 3D stack. This 2D slice shows some 53BP1 IRIF where the corresponding pDNA-PKcs IRIF is in another slice above or below. The larger 53BP1 IRIF contain two or more pDNA-PKcs IRIF. (**b**) IRIF size determined for each dose and time point. (**c**) The upper panel shows the number of IRIF per cell for 53BP1 (black) and pDNA-PKcs (red) for each dose and time point. The lower panel depicts the corresponding nuclear volume. The radiated samples show significantly more IRIF per cell, but no significant difference is visible for the irradiation geometries. The raw data box plots can be found in Supplementary Fig. 2. Cell volume measurements reveal that a large difference can be seen between the radiation geometries. Therefore the pure counting is not feasible in this case. (**d**) IRIF density for γ-irradiation over time. For all time points, the IRIF density is significantly higher for 3.0 Gy than after 1.9 Gy irradiation. Throughout the whole period, the number of 53BP1 and pDNA-PKcs IRIF do not differ significantly for both irradiation doses but are slightly higher for pDNA-PKcs at all times (**e**) mean IRIF density over dose.

First, it is proven that pDNA-PKcs works as a biodosimetry marker for low-LET radiation. Commonly, γH2AX and 53BP1 are used for dosimetry in this LET regime. But a marker for high-LET dosimetry must unequivocally also serve as a dosimetry marker for low-LET, otherwise, a quantitative measurement in the high-LET regime may not be viable. Thus, it was tested whether pDNA-PKcs labels every DSB after low-LET reference radiation just as it is assumed for γH2AX and 53BP1. The labeling of pDNA-PKcs was compared to 53BP1 labeling after ^60^Co γ-irradiation. The samples were irradiated with two different doses of (1.9 ± 0.2) Gy and (3.0 ± 0.3) Gy. The doses were chosen to ensure that the damages are still well separated in the cell nucleus and thus proper counting of 53BP1 IRIF is possible. Double labeling of 53BP1 and pDNA-PKcs showed that all 53BP1 IRIF contain at least one pDNA-PKcs IRIF, as shown in Fig. 1a and quantified in Fig. 1c,d for several time points after irradiation. For counting only pDNA-PKcs IRIF were used, which colocalize with a 53BP1 IRIF. This condition that an IRIF is only counted if it is double positive is a method also used in conventional γH2AX and 53BP1 assays to make the damage assessment more reliable. Unirradiated control cells showed almost no foci (cf. Supplementary Fig. 1), which proves that the counted foci are true ionizing radiation-induced foci for both proteins. Therefore one can conclude that all additional co-stained foci are due to radiation and can be valuable for counting and dosimetry.

Figure 1a clearly shows pDNA-PKcs foci are much smaller than 53BP1. The IRIF size was measured using autocorrelation of fluorescence images from STED microscopy^16^ to determine the FWHM for each pDNA-PKcs IRIF. The size of the pDNA-PKcs IRIF was measured as (140 ± 20) nm (FWHM), which was well above the 100 nm resolution as obtained for STED microscopy under the settings used here, the detailed size distribution can be found in Fig. 1b. The size of the 53BP1 IRIF was determined to be (600 ± 40) nm. Some of the larger 53BP1 IRIF contain two or more pDNA-PKcs IRIF, indicating that there may be two or even more DSB inside. The multiple pDNA-PKcs IRIF within some of the individual 53BP1 IRIF causes the slightly enhanced numbers of total pDNA-PKcs foci within the irradiated cells (Fig. 1c–e, see also Table 1).

**Table 1:** IRIF numbers, cell volume and analyzed number of cells for 1.9 Gy and 3.0 Gy of gamma irradiation for all time points. Uncertainties represent the standard error of the mean.

Dose	Time (min)	53BP1		DNA-PKcs		Cell volume (µm^3^)	Number of cells
		#IRIF/cell	#IRIF/µm^3^	#IRIF/cell	#IRIF/µm^3^		
0 Gy		5 ± 4	0.007 ± 0.006	4 ± 4	0.006 ± 0.006	723 ± 100	20
1.9 Gy	3	32.7 ± 2.6	0.039 ± 0.004	35.4 ± 2.5	0.043 ± 0.006	864 ± 55	14
	5	29.7 ± 3.7	0.044 ± 0.005	35.9 ± 4.8	0.052 ± 0.005	685 ± 79	14
	10	30.5 ± 3.6	0.042 ± 0.009	35.1 ± 5.1	0.0486 ± 0.011	843 ± 108	20
	30	27.7 ± 2.8	0.0292 ± 0.0018	29.3 ± 2.5	0.0312 ± 0.0013	964 ± 111	18
3.0 Gy	3	39.5 ± 2.5	0.060 ± 0.004	43.1 ± 2.5	0.064 ± 0.003	675 ± 57	18
	5	35.0 ± 3.0	0.073 ± 0.009	35.3 ± 2.9	0.073 ± 0.009	531 ± 65	20
	10	33.3 ± 2.5	0.060 ± 0.005	35.2 ± 2.4	0.064 ± 0.005	567 ± 36	20
	30	39.2 ± 4.8	0.062 ± 0.006	39.9 ± 4.7	0.063 ± 0.006	660 ± 91	18

The number of IRIF was counted for both damage markers 3, 5, 10, and 30 min after irradiation. For each time point and irradiation dose, at least 14 cells from two independent experiments and three samples per experiment were analyzed (see Table 1). The nucleus volume and therefore DNA content greatly varies between single HeLa cells (Fig. 1c). To obtain an unbiased, comparable value, the number of IRIF per nucleus is divided by the corresponding nuclear volume. This gives the normalized IRIF density which is a comparable measure for a cell line with greatly varying DNA content as is the case for HeLa cells (Fig. 1d). For pDNA-PKcs, 0.043 IRIF/µm^3^ for 1.9 Gy and 0.064 IRIF/µm^3^ for 3.0 Gy were measured 3 min after irradiation. For both doses, although a slight increase of the IRIF numbers is visible after 5 min, the IRIF density is constant within the measurement uncertainty in the first 10 min after irradiation. For 1.9 Gy the value decreases towards 30 min, whereas for 3.0 Gy it stays constant. Using the first 10 min as a reliable measure for the non-processed, radiation-induced damages, i.e. averaging the 3, 5, and 10 min time points, gives mean numbers of pDNA-PKcs foci of (0.048 ± 0.008) IRIF/µm^3^ for 1.9 Gy and (0.067 ± 0.007) IRIF/µm^3^ for 3.0 Gy (cf. Fig. 1e).

The almost perfect one-to-one correlation of pDNA-PKcs with 53BP1 in combination with the small IRIF size beyond the resolution limit of conventional microscopy allows to use pDNA-PKcs as proper labeling for DSB and thus for dosimetry purposes already in the first minutes after irradiation. In particular, dosimetry comes into reach even for high LET radiation. The tiny pDNA-PKcs IRIF can be separated at high local DSB densities where the large 53BP1 IRIF overlap and limit a retrospective dose evaluation.

### pDNA-PKcs IRIF counting after high-LET particle irradiation

**Figure 2:**
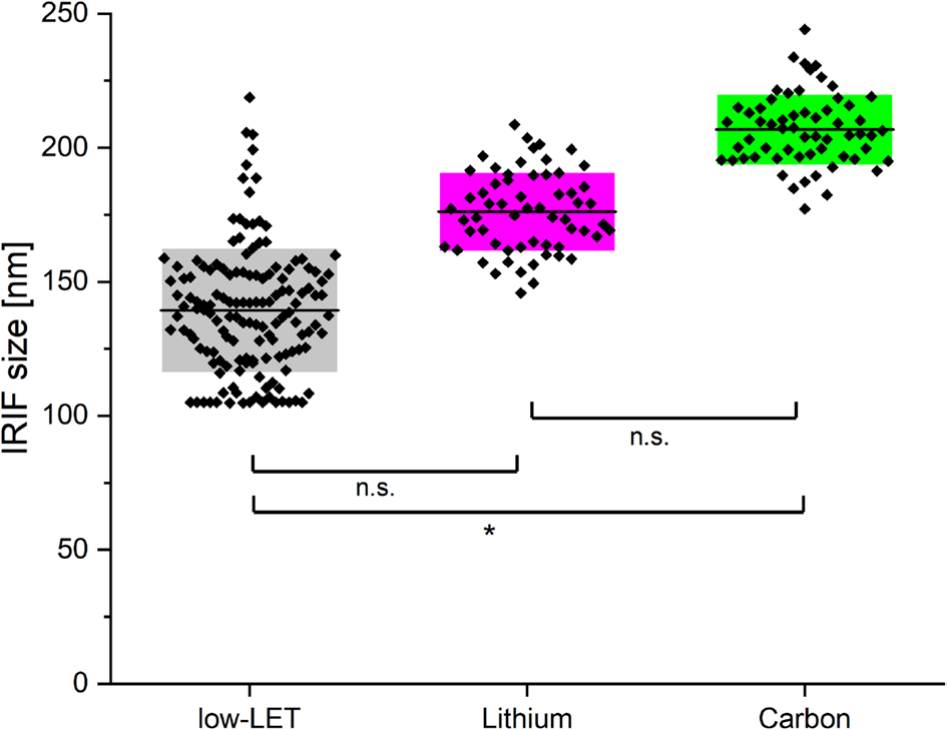
Comparison of IRIF sizes for low-LET photon (black) irradiation, as well as lithium (magenta) and carbon (green) ion irradiation.

Human HeLa cells were irradiated at the ion microprobe SNAKE with high-LET carbon (D_mean_ = 1.2 Gy, E = 27 MeV, LET = 500 keV/µm) and lithium ions (D_mean_ = 0.2 Gy, E = 20 MeV, LET = 116 keV/µm). The cells were fixed and labeled for 53BP1 and pDNA-PKcs 3 min, 5 min, and 10 min after irradiation. The IRIF size was again measured using the autocorrelation function to determine the FWHM for each pDNA-PKcs and 53BP1 IRIF. For 53BP1 the IRIF size was (640 ± 40) nm, comparable to the size of the IRIF after photon irradiation and the same for both ion species. The pDNA-PKcs IRIF size is (175 ± 14) nm for lithium irradiation and (205 ± 15) nm for carbon irradiation, as depicted in Fig. 2. Although the increase in size between the two ion species is not statistically significant, a trend to bigger IRIF size with increasing LET is visible, especially when comparing the sizes to the low-LET IRIF size of (140 ± 20) nm. The difference between photon and carbon ion-induced IRIFs is statistically significant (t-test, p < 0.05).

The bigger IRIF size can be explained by the fact that with increasing LET the local concentration of DSB is increasing and therefore the number of pDNA-PKcs at the damage location. Furthermore, increased signal accumulation in 3D can lead to increased size in the 2D imaging and measurement. Both lead to increased measured IRIF sizes. The size of the photon-induced IRIF can be considered to be the accumulation for a single DSB. The higher the LET the more DSB are located within one pDNA-PKcs IRIF.

**Figure 3:**
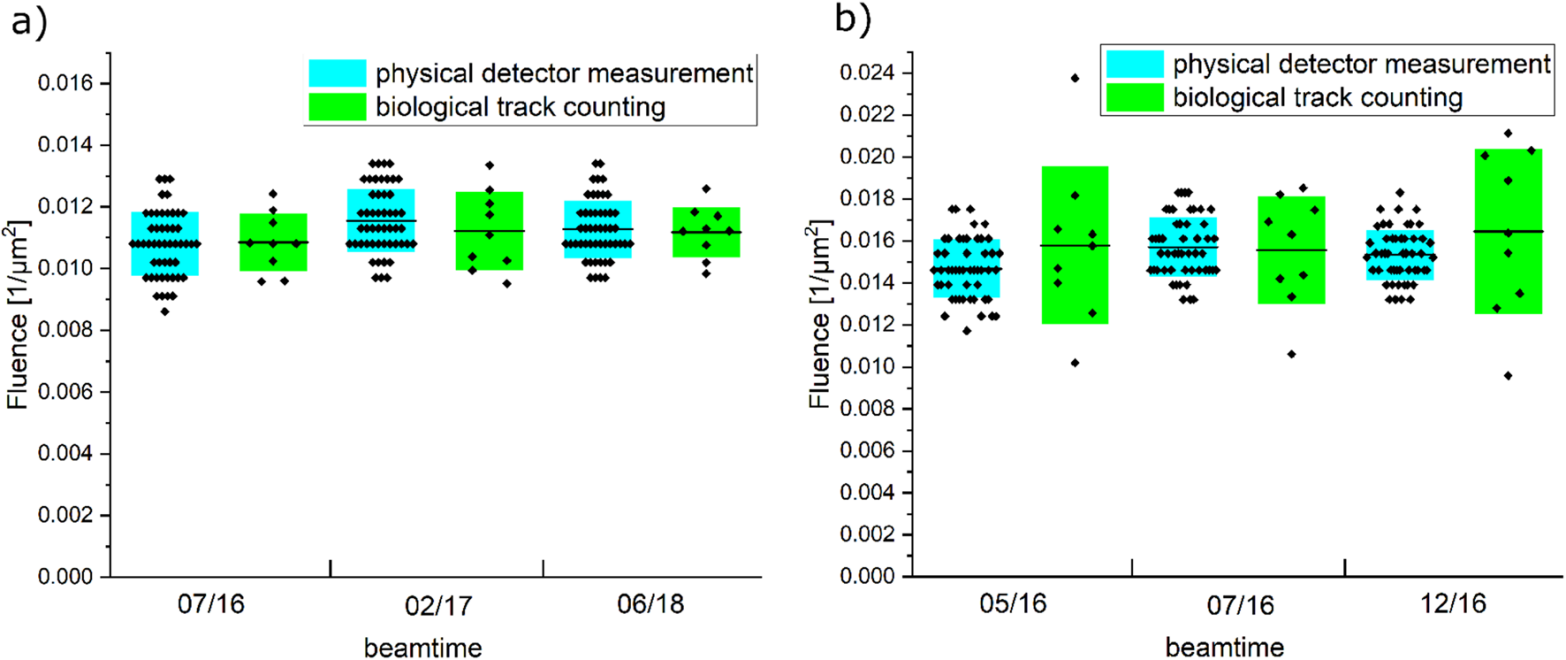
Comparison of the fluence measurements for (**a**) lithium ions and (**b**) Carbon ions for each beamtime. The physical detector measurements are shown in cyan and green the biological track counting. No statistically significant difference between the measurements for each ion could be detected.

Furthermore, the sensitivity of the IRIF assay was determined, by comparing the ion fluence determined from the cell measurements and the ion fluence coming from detector measurements. For high-LET particles the fluence in a cellular system can be measured as the mean number of tracks per cell nucleus divided by the nuclear area, resulting in $\text{F}=\frac{\#\text{ tracks}}{{\upmu}{\text{m}}^{2}}$. For each ion species, this was done for 900 cells in total for nine samples per beam time. To test the sensitivity this needs to be compared to the fluence measured with the ion detector. The mean fluences are depicted in Table 2 and the fluences measured per beamtime are shown in Fig. 3, no statistically significant difference in the fluences measured in the cells and coming from detector measurements can be found.

**Table 2:** Fluence for carbon and lithium ion irradiation determined either from the cells or by the PMT detetctor.

Ion species	Fluence from cells (/µm^2^)	Fluence from detector (/µm^2^)
Carbon	0.016 ± 0.003	0.015 ± 0.002
Lithium	0.010 ± 0.002	0.011 ± 0.002

**Figure 4:**
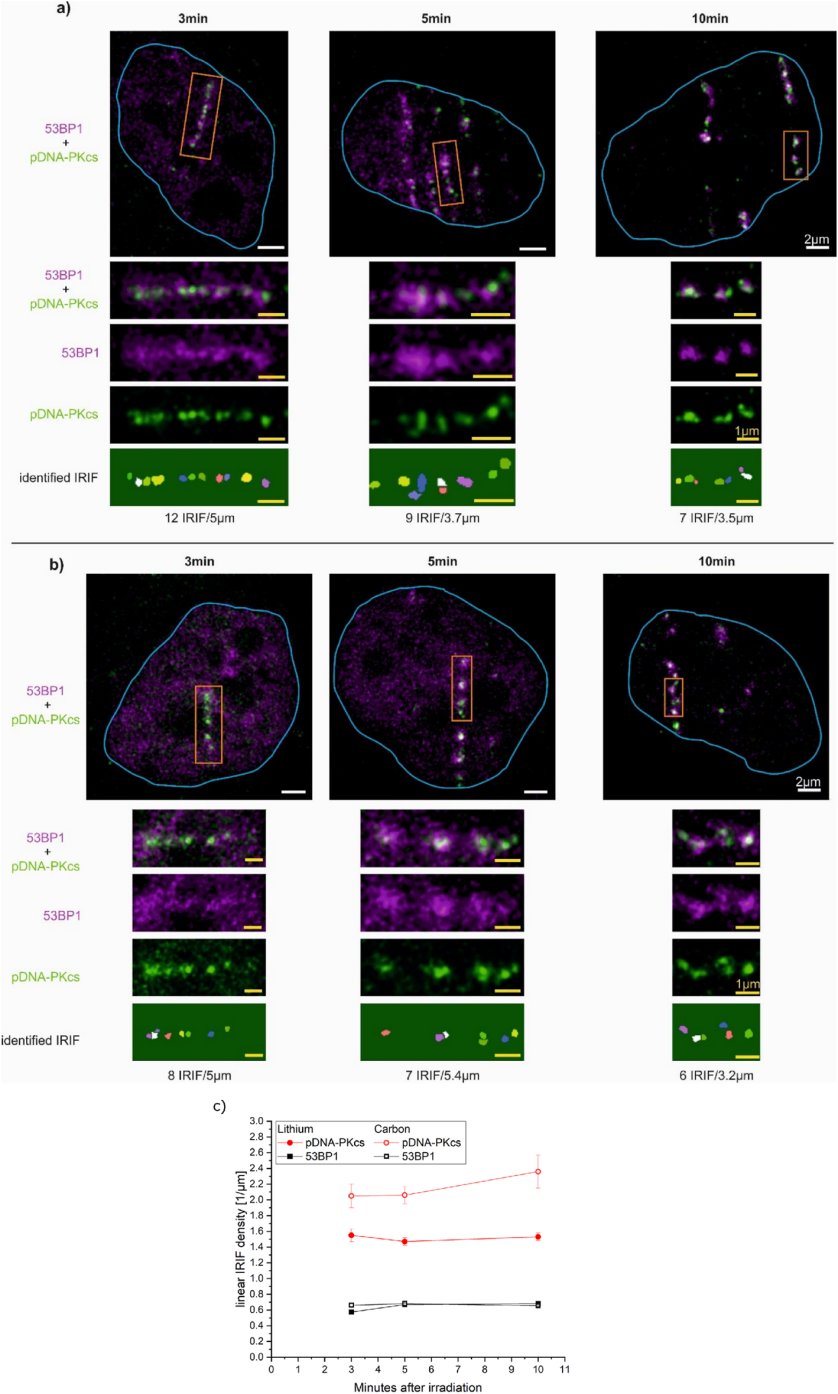
HeLa cells irradiated with (**a**) carbon and (**b**) lithium ions 3 min, 5 min, and 10 min after irradiation. Each track represents one ion traversal. The orange boxes are enlarged in the images below. The last row shows the identified IRIF and the number of IRIF and measured track length. (**c**) Linear IRIF density of pDNA-PKcs IRIF (red) and 53BP1 IRIF (black) 3 min, 5 min, and 10 min after carbon (open symbols) and lithium (filled symbols) irradiation.

After being convinced that the sensitivity of the assay is sufficient the counting of the IRIF is performed for high-LET irradiation. Furthermore, the linear IRIF density, i.e. the number of IRIF per µm track length, along the ion traversal is determined. As for low-LET irradiation, the samples are double-stained for pDNA-PKcs and 53BP1, only IRIF located in the double-positive region are counted. Sample images for carbon and lithium-ion irradiation, where the imaging plane is tilted by 9° compared to the irradiation direction, are displayed in Fig. 4a,b, respectively.

The number of IRIF was determined using the FociPicker plugin^21^ for ImageJ, the corresponding IRIF mask for pDNA-PKcs is depicted in the last row in Fig. 4a,b. This plugin can identify separate clusters by intensity separation of intensity minima and maxima, taking also 3D information into account. The mean numbers of IRIF at 3 min, 5 min, and 10 min after irradiation are depicted in Fig. 4c and were each determined from 20 cells from three independent experiments for both carbon (with 1–3 tracks per cell) and lithium (1–5 tracks) ion irradiations (see Supplementary Table 1). For 53BP1, the difference in the density could not be measured either in a time or ion species-dependent manner. The mean 53BP1 LID for lithium-ion irradiation is (0.64 ± 0.12)/µm and for carbon ion irradiation is (0.67 ± 0.11)/µm within 10 min after irradiation. Carbon ion-induced damages show more pDNA-PKcs IRIF along a certain track length (Fig. 4c, red, open symbols) compared to the lithium ion-induced damages (Fig. 4c, red, filled symbols) at all time points. For lithium-ion irradiation, the linear IRIF density is slightly decreasing with increasing time, whereas for carbon ion irradiation the density is slightly increasing with increasing time. However, for both ion species, the changes are not significant and thus are considered to remain constant for both ion species. Thus, mean pDNA-PKcs LID of (2.2 ± 0.2) IRIF/µm for carbon ions and (1.5 ± 0.1) IRIF/µm for lithium ions are used.

### Simulation of DSB induction and linear density of DSB clusters

**Figure 5:**
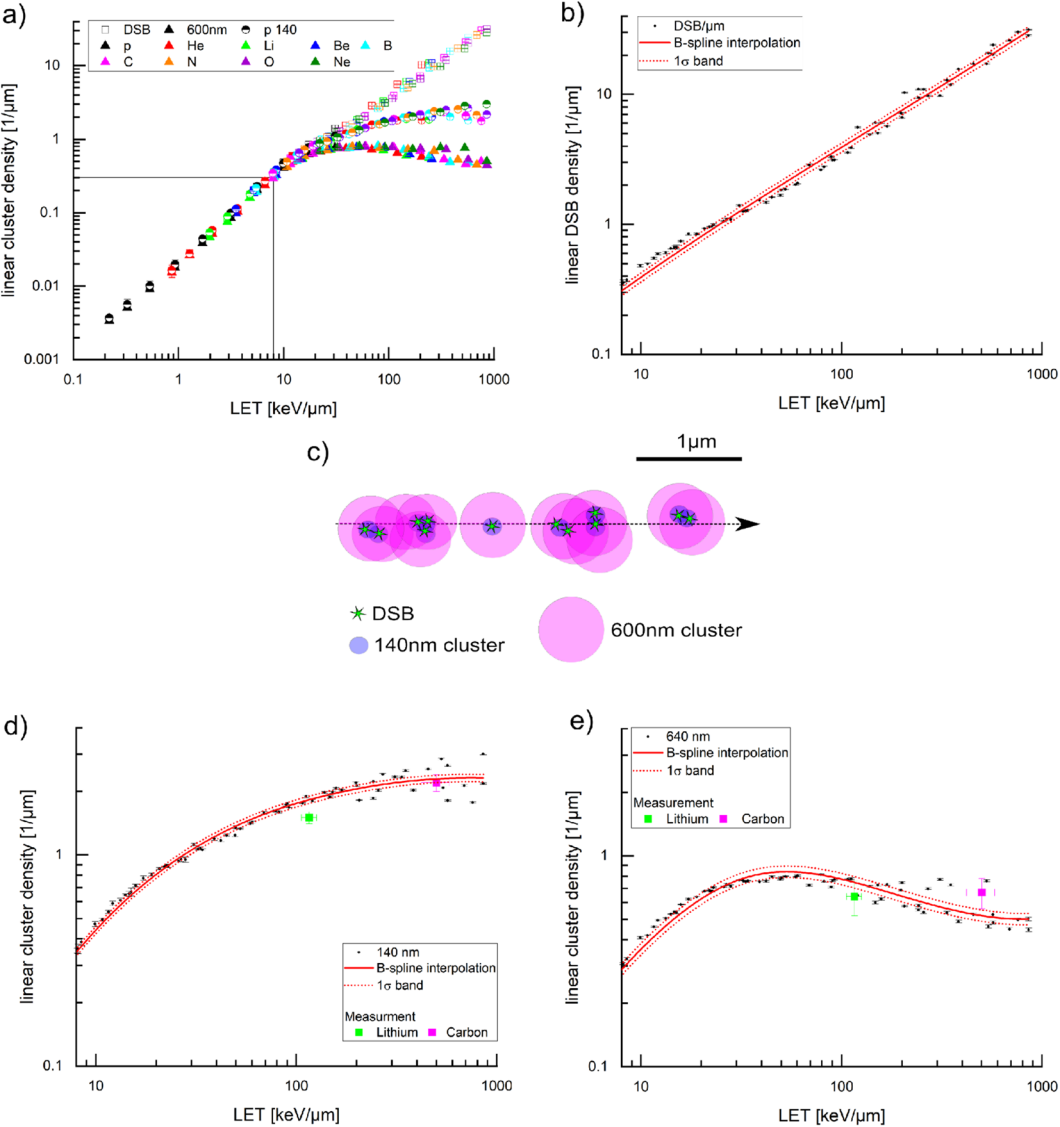
(**a**) Linear DSB density (empty squares) from PARTRAC simulation for nine different ions (hydrogen, helium, lithium, beryllium, boron, carbon, nitrogen, oxygen, and neon) over a LET range from 0.2 to 860 keV/µm. 140 nm clusters (semi-filled circles) and 600 nm clusters (filled triangles) from PARTRAC simulated DSB clusters with the method depicted in (**c**). Black lines give the lower termination criterion. (**b**) Linear DSB density with B-spline (red line) and the corresponding 1σ confidence band (dashed, red line). (**c**) Visualization of the method of clustering the DSB from the simulation. Green stars represent 12 DSB. The violet circles are the six clusters with 140 nm and the pink circles are the 640 nm clusters. (**d**) Linear cluster density for 140 nm (**d**) and 640 nm (**e**) cluster width with B-spline (red line) and the corresponding 1σ confidence band (dashed, red line). The measured data for pDNA-PKcs are shown for both lithium (green) and carbon ion (magenta) irradiation. In (**d**) and (**e**), the y-axis is zoomed in to a maximum value of 4 µm^−1^.

To investigate if the number of pDNA-PKcs IRIF corresponds to the number of induced DSB for high LET irradiation (i.e. LET > 10 keV/µm), it is not possible to use experimental data since there is no way to directly measure the total DSB numbers or, even more demanding, their local distributions. Therefore, we used the simulated DSB distributions from PARTRAC^19^, which uses experimental and theoretical information for all physical, chemical, and biological processes of DNA damage induction by energetic ions, and a detailed model of the chromatin structure of human cells. In this simulation, a DSB is formed when two breaks in the opposite strands of the DNA molecule occur within ten base pairs. Additionally, a probability of 1% is used for the conversion of a single strand break to a DSB. This simulation gives the local damage induction along and radial to the ion traversal. Consequently, the location of each DSB is obtained within this model and is used to calculate the linear DSB density, i.e. the number of DSB per µm track length. Figure 5a (empty squares) shows the simulated linear DSB density for nine different ion species, namely proton, helium, lithium, beryllium, boron, carbon, nitrogen, oxygen, and neon at different energies covering a LET range between 0.2 and 860 keV/µm. As abort criterion for maximum LET of these ions, the condition was used that the residual energy is sufficient such that the ions can traverse a whole cell nucleus of 10 µm diameter. The linear DSB density is nearly independent of the ion species but mainly depends on the LET of the ions.

For decreasing LET the linear DSB density is decreasing. At the lower LET region, the probability to obtain a DSB from a single ion is below one. To identify irradiations from high LET particles in an experiment it is crucial to be able to identify the tracks as such. We, therefore, define 0.3/µm as a lower limit of linear density, i.e. three clusters in a cell nucleus of 10 µm diameter, to identify a high LET ion transversal. This limit turns out to be at a LET of 8 keV/µm. Thus, any kind of high LET particle can be detected by track recognition according to this limit since a LET > 10 keV/µm is commonly considered as high LET.

In Fig. 5b the linear DSB density is plotted with a lower LET limit of 8 keV/µm and an upper limit of 860 keV/µm and no differentiation is made between different ion species. The data show a linear relationship over the whole LET range, visualized by a B-spline interpolation together with a 1σ confidence band, which contains 68% of all data points.

In a further step the linear DSB density is turned into a linear cluster density (LCD) that can be compared to the LID of measured IRIFs, which is limited by the IRIF size of a single DSB of 140 nm for pDNA-PKcs and 600 nm for 53BP1. The coordinates of each DSB that is given from the simulation are used to group DSB into clusters for a given cluster size. Thus, the clusters are the simulation counterpart of the measured IRIF. The cluster sizes of 140 nm and 600 nm are used for comparison corresponding to the high-LET pDNA-PKcs and 53BP1 IRIF respectively. The method of clustering the simulated DSB distributions is shown in Fig. 5c. The location of the first DSB along the simulated ion traversal is used as starting point for the first cluster. As long as a second DSB is within the given finite distance of 140 nm or 600 nm, it is taken within the first cluster. This procedure is then performed with all DSBs until no further DSB is found within the finite distance to one of the DSBs already present in the cluster. Then, the location of the next DSB which is not part of the cluster is searched along the ion track, where the next cluster is started (cf. Fig. 5c). The procedure is performed until each DSB is part of a cluster. With this method, it is possible to trace the distribution of clusters of different sizes, which can then be compared to the measured IRIF distribution for sizes larger than the microscopic resolution. Further visualization of the method can be found in Supplementary Fig. 3.

Figure 5a shows the corresponding data of linear cluster density over LET for 140 nm (semi-filled circles) and 600 nm (filled triangles) in comparison to the linear DSB density. For LET values below ~ 10 keV/µm, all three curves overlap. This is the region where the number of IRIF or clusters represents the DSB numbers according to the simulation. When performing an IRIF counting measurement in this LET range the number of IRIF is expected to correspond well to the number of induced DSB. With increasing LET this direct one-to-one correlation is lost. The cluster curves start to bend and are approaching a maximum value of LCD. For the 600 nm clusters, this starts earlier than for the 140 nm clusters and the maximum LCD is also lower. Again, the linear cluster density is almost independent of ion species but strongly dependent on LET beside large LET values where the LCD-LET dependence becomes flat.

The linear cluster density for 140 nm clusters is shown in Fig. 5d. The data were interpolated using a B-spline interpolation and is shown together with the 1σ confidence band. The spline shows a linear increase up to a LET value of ~ 15 keV/µm, for higher values the curve starts to bend but still a monotonic increase is visible approaching a maximum LCD of (2.31 ± 0.09)/µm. A real increase of the linear cluster density is obtained up to a LET of ~ 300 keV/µm, for higher LET values the curve can be considered constant.

For 600 nm the LCD starts to bend already at a LET of ~ 8 keV/µm. The data together with the B-spline interpolation are shown in Fig. 5e. A monotonic increase is achieved up to a LET of 35 keV/µm. After that a plateau region extends for higher LET with a maximum LCD = (0.84 ± 0.04)/µm. Interestingly, for even higher LET values > 80 keV/µm the curve even decreases again.

### Comparison of measured data and simulation

Linear cluster densities from PARTRAC data are compared to the measured LID for the 600 nm sized 53BP1 IRIF (Fig. 5e). The two measured linear IRIF densities of lithium and carbon tracks coincide within the error bars with the interpolated LCD and are well represented by the PARTRAC data. For comparison with simulation, the measured linear pDNA-PKcs IRIF densities are compared to the LCD values for the 140 nm cluster in Fig. 5e. The lithium measurement results in a LID = (1.5 ± 0.1)/µm at a LET of (116 ± 10) keV/µm. The expectation from the simulation at this LET is (1.83 ± 0.10)/µm. Thus the experimental value lies below the expectation from simulation considering the 1σ confidence band. For carbon, the experimental value is LID = (2.2 ± 0.2)/µm at an LET = (500 ± 80) keV/µm. Simulation gives a value of LCD = (2.29 ± 0.12)/µm. Here the measured value lies within 1σ confidence band of the simulation fit.

### Dosimetry of high-LET particle irradiation in human HeLa cells

**Figure 6:**
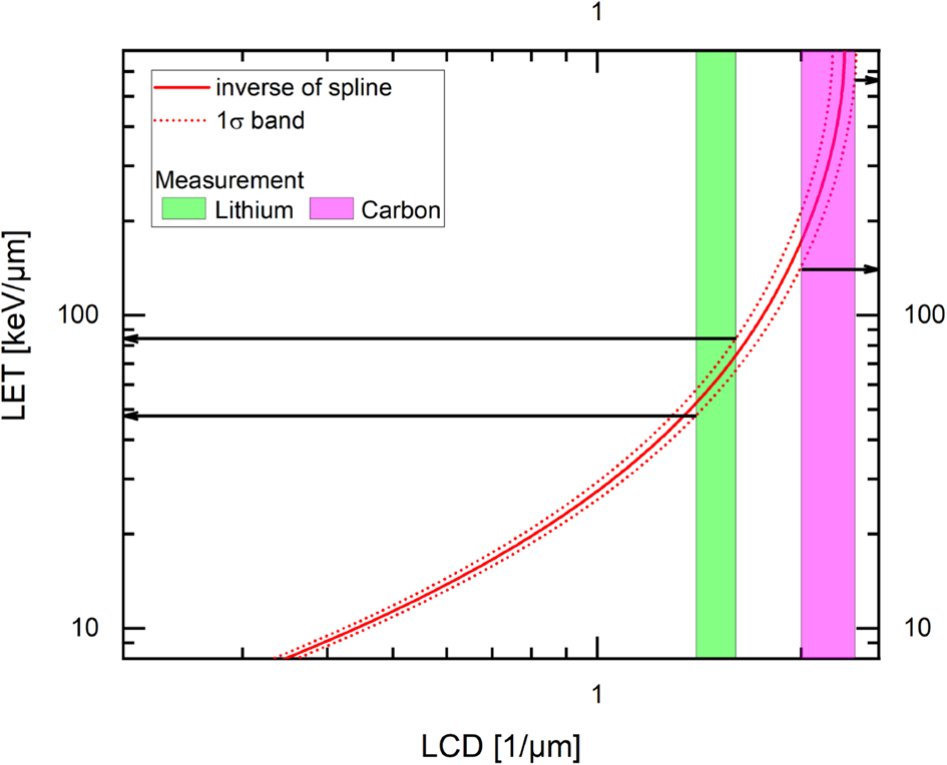
Inverted spline curve of LET for dosimetry. The values of LID for lithium (green) and carbon ion (magenta) irradiation are added as boxes to be able to determine LET (from the intersection of box and fit).

The linear IRIF density can be used as a measure for LET, as both quantities show a monotonical relationship. The smaller the IRIF size the higher LET values can be evaluated. Although an IRIF may contain two or more DSB and thus the linear relationship declines at higher LET values, the monotonic increase allows for an inversion of the LCD to LET relationship up to a certain LET value given by the IRIF size. In the case of 600 nm sized 53BP1 IRIF LET values of only up to 80 keV/µm can be evaluated while with the much smaller pDNA-PKcs IRIF up to a LET of 240 keV/µm can be rated. Above this value, the increase in linear IRIF density is too low to obtain reliable LET values from a measured linear IRIF density. Thus, LET can be determined from linear cluster density up to this maximum LET value. Ideally, the dosimetry would rely on experimental data where the LET relationship to linear IRIF density and the IRIF size is determined for different ion species with different energies. Establishing such a database is an extreme effort and waits for a large set of experiments. To show the principle options for the inversion of LET to linear IRIF density the simulated linear cluster density data are used instead. To be able to perform dosimetry using the pDNA-PKcs labeling the simulation for 140 nm resolution is used here. LET can be determined from LCD according to the simulated data by just inverting the spline (Supplementary Table 2) or using the graphical representation in Fig. 6.

The dose and the LET are linked via the formula: $\text{D}=\frac{\text{F}\times \text{LET}}{\uprho }$, where ρ is the density of water and F the particle fluence. Therefore, dosimetry can be performed when the LET and particle fluence F are known. The LET is measured using the comparison of LID with LCD. For irradiation with particles with a single LET, as done in the experiments in this study, the product of fluence and LET defines the dose. For exposure with mixed particles of varying LET, the dose is calculated as

$$D=\frac{1}{\rho }\sum_{i=1}^{n}{F}_{i}\times {LET}_{i},$$

where *i* is ranging from 1 to *n,* for *n* different kinds of particles.

As the first proof of principle, biodosimetry is performed for HeLa cells irradiated with lithium ions in this study. To determine the LET from the measurement, LID = (1.5 ± 0.1)/µm is compared with the function for LET(LCD) resulting from simulation. It is determined at which LET the number of clusters corresponds to the measured LID. Using this method, a LET of (66 ± 19) keV/µm is measured. For lithium-ion irradiation, a fluence F of (0.011 ± 0.001)/µm^2^ was determined as described above. Thus, the dose and the dose error can be calculated:

$$ D_{measured, Li} = \frac{F \times LET}{\rho } = \frac{{0.011\;\frac{1}{{\upmu {\text{m}}^{2} }} \times 66\;\frac{{{\text{keV}}}}{{\upmu {\text{m}}}}}}{{1000\;\frac{{{\text{kg}}}}{{{\text{m}}^{3} }}}} = 0.12\;{\text{Gy}} $$

$$ \begin{aligned} \Delta D_{measured,Li} &= \frac{LET}{\rho } \times \Delta F + \frac{F}{\rho } \times \Delta LET \\ & = \frac{{66\;\frac{{{\text{keV}}}}{{\upmu {\text{m}}}}}}{{1000\;\frac{{{\text{kg}}}}{{{\text{m}}^{3} }}}} \times 0.003\;\frac{1}{{\upmu {\text{m}}^{2} }} + \frac{{0.010\;\frac{1}{{\upmu {\text{m}}^{2} }}}}{{1000\;\frac{{{\text{kg}}}}{{{\text{m}}^{3} }}}} \times 19\;\frac{{{\text{keV}}}}{{\upmu {\text{m}}}} = 0.06\;{\text{Gy}} \\ \end{aligned} $$

This dose (D_measured,Li_ = (0.12 ± 0.06) Gy) can be compared to the actual irradiated mean dose as defined by the ion fluence measured on a photomultiplier detector and the calculated LET of (116 ± 10) keV/µm (D_PMT,Li_ = (0.20 ± 0.05) Gy). Due to the underestimation of the LET the measured dose is below the dose detected using the photomultiplier. Nevertheless, the values are coinciding within the uncertainties.

## Discussion

We presented a new method for dosimetry in human cells using counting of pDNA-PKcs IRIF, offering for the first time the possibility to perform quantitatively, retrospective dosimetry for high-LET particle exposure and prove this method on experimental data. DNA-PKcs is the catalytic subunit of the DNA protein kinase, which is an early responder to DSB^25^. Phosphorylation of DNA-PKcs on the ABCDE cluster containing the T2609 site promotes DNA-PKcs release but also marks DNA damage site by forming radiation-induced foci of small size for single DSB of (140 ± 20) nm in the first 30 min after irradiation. This focus size is about 4-times smaller than for commonly used 53BP1 and gH2AX IRIF. The early clustering at every DSB forming small foci makes the phosphorylated form of DNA-PKcs (termed pDNA-PKcs) a perfect candidate for dosimetry purposes. First, we proved this by showing that it exhibits a one-to-one correlation with 53BP1, a well-established marker used in biological dosimetry^19,21^, for low-LET γ-radiation.

For high-LET measurements, we first checked if the sensitivity of the IRIF assay is sufficient by comparing the determined ion fluence from the biological samples with the ion fluence coming from the detector measurements. The particle fluence F (#tracks/µm^2^) in the biological sample can be defined by counting the number of tracks per nucleus divided by the nucleus size. No difference between the two measurement methods, either track counting in the cells or detector measurements, was visible. We conclude that every ion, which hits the cells can be detected with the method used.

We then used the pDNA-PKcs IRIF labeling together with 53BP1 on high-LET particle tracks after lithium-ion irradiation (LET = (116 ± 10) keV/µm) and carbon ion irradiation (LET = (500 ± 80) keV/µm) to perform dosimetry in the cells. The LET is defined by the linear IRIF density calibrated using PARTAC simulation^40,42^. According to the formula $D=\frac{LET\times F}{\rho }$, the dose can be calculated. The calibration has to be performed using simulated data, as no experimental database is existing. Calibration to experimental data would be preferable. Creating such a database is an effortful project, therefore we decided to first show a proof-of-principle using a well-accepted simulation tool. Monte Carlo PARTRAC simulations^43^ gives the number of DSB per µm track length dependent on LET for various kinds of particles^40,42^. This simulation shows a monotonically increasing relationship between DSB/µm and LET, which is independent of particle type. However, by using the methods of counting fluorescently labeled protein IRIF the exact number of DSB cannot be counted, due to the finite size of the IRIF. Therefore, we used the location of the DSB given by the simulation and clustered them to finite clusters of sizes, which correspond to the measured IRIF sizes. The clustering leads to overall reduced numbers compared to actual induced DSB but still shows a monotonic increasing relationship, which is the necessary criterion for dosimetry. But now, this relationship is only valid up to a certain maximum LET, which is defined/determined by the cluster size. This signifies that the larger the cluster size the lower the maximum LET up to which dosimetry is possible. We proved this method using our data on pDNA-PKcs for lithium as well as carbon ions. For lithium, the determined LET was LET_Li,pDNA-PKcs_ = (66 ± 19) keV/µm. Using the counted fluence of F = (0.011 ± 0.001)/µm^2^ the corresponding doses are D_Li,pDNa-PKcs_ = (0.12 ± 0.06) Gy. The dose was determined using the count rate of the photomultiplier detector and the SRIM-calculated LET is D_PMT,Li_ = (0.20 ± 0.05) Gy. First of all, one sees that with the proposed method, although well better than with conventional 53BP1 counting, the LET and therefore the dose is underestimated. In contrast, fluence measurements work well. The reason for the underestimation of LET needs to be investigated in detail in the future. Some possible explanations shall be named here. First, the use of simulation data as a data basis could hold uncertainties. Therefore it would be better to use an experimental database, also because in measured data the change in size is inherently included and errors due to size under- or overestimation can be excluded. A further reason could lie in the use of HeLa cells in this first proof-of-principle study. HeLa cells are highly aneuploid and therefore the linear DSB density can vary due to higher or lower DNA content. Additionally, for the use in biodosimetry, the cell cycle needs to be taken into account, as this also influences the linear DSB density. Nevertheless, already with this non-optimized measurement as a first-proof-of-principle the dosimetry works quite well, as both the physically and biologically determined doses coincide. One has to note that also the physically determined dose has a rather large error, which comes from unpredictable beam current fluctuations. Furthermore, the used LET is based on simulations using the SRIM program rather than from real measurements. This can be an additional source of uncertainty.

We conclude that for exposure with a known ion type this new method of dosimetry works in a very large LET range up to 240 keV/µm. This is a region where conventional retrospective biodosimetry methods fail. The presented proof-of-principle study may suffer from the problem that calibration data from PARTRAC simulation were used and adapted for analysis. This might result in too low cluster numbers and therefore lead to an underestimation of LET. For future experiments a database from experimental measurements is necessary.

As for finite cluster sizes, at the plateau in the relationship between cluster/µm and LET, the dosimetry of unknown particles gets to a limit. As long as the particle LET is in the monotonically increasing region, dosimetry works well. The particle type does not have to be known, which is a clear advantage. For LET values > 240 keV/µm, the dosimetry of unknown particles would, at the moment, fail. A possibility to solve this problem is to go to even smaller labeling, using proteins such as Ku70/80, which is located as a heterodimer at the damage and has the potential to give incredibly small IRIF. Until now, for fluorescence microscopy this is challenging, as bright and stable labeling has to be established, withstanding the high laser intensities, which are necessary to get the desired resolution around 100 nm. The ideal labeling would be the direct labeling of DSB with a fluorescent tag, which is until now not possible for STED microscopy but has to be developed in the future. Another advantage of the presented method of retrospective biodosimetry is that it can be used on single cells, which makes it a quick but still accurate assay. Furthermore, this method can also be used for mixed particle exposure of high-LET as for each ion track the number of IRIF/µm and the corresponding fluence can be determined and the single doses can be just added up to the total dose. Moreover, we proved that pDNA-PKcs labeling can also be used for low-LET irradiation, which opens for the first time the possibility to perform biological dosimetry on mixed field radiation.

This method brings big advantages to several fields of research and radiation dosimetry. It can give information on the real biological mechanisms in modern tumor therapy approaches such as e.g. particle therapy, where high-LET particles are used, and boron-neutron or boron-proton capture therapy, where both low- and high-LET particles damage the cells. This understanding helps to improve the effectiveness of these therapy approaches. Furthermore, the exposure of astronauts on space missions can be analyzed in detail. This can help to develop radiation safety concepts for deep space missions such as the mission to Mars. Last, this method can be used for dosimetry in radiation accidents, e.g. at particle therapy centers or nuclear power plants. These are only three examples of a large field of applications that can be imagined. Nevertheless to be able to transfer this method into daily use further investigations need to be performed to overcome current limitations. First of all, biodosimetry conventionally is performed in the lymphocytes gathered from blood samples, so a first step will be to prove the dosimetric potential of the proposed method in different cell types, especially the lymphocytes. This method in principle has the advantage that it can be transferred to non-adherent cell lines such as lymphocytes. Therefore it is preferable compared to pre-extraction-based methods imaging DNA-PKcs or Ku70/80 directly^35^ as the pre-extraction inherently excludes the use on these cells. Furthermore, it could be imagined to find better-suited labels for double-strand breaks such as the direct label of Ku70/80 as done before for super-resolution STORM microscopy^33,34^. A careful evaluation of the optimum configuration needs to be done in the future.

Furthermore, a larger study has to be established also testing different ion types and doses, which is also needed to set up the database necessary for dosimetry of unknown exposure. The main limitation of this method at the moment is that the cells which should be analyzed have to be collected and fixed a few minutes after exposure. This is feasible for research purposes and also in space flights, where astronauts could take and analyze blood samples constantly during their flight. But in other applications, such as retrospective measurements after a radiation accident, this is not easily achievable. Therefore the method needs to be extended to later time points up to one day or even longer. The applicability of the use of pDNA-PKcs for these later time points needs to be investigated with care in further studies. One has to note that only collection and sample processing have to be performed fast as the samples can be stored up to several weeks before labeling and super-resolution imaging has to be performed. In contrast, a major advantage is that in total quick analysis lasting no longer than 24 h in total is possible, when labeling and imaging are performed just after fixation.

The biodosimetric measurement of partial body exposure gets an important point in the research for high-LET particle exposure. A small number of particles already give a significant amount of dose to the exposed individuum. The biodosimetry using blood samples might underestimate the dose as less irradiated cells might be collected. Our method is bound to the same limitations as all other assays are, but together with the simulations research can be performed to improve the biodosimetry in this direction.

We conclude that this new method poses the potential to be a versatile, quick, but very accurate way of doing biological dosimetry on all kinds of particle exposure. This is the first time that this is possible and it is needed in various kinds of applications ranging from medical and space research to the use as a dosimetry method for radiation safety.

## Methods

### Cell cultivation and immunofluorescence detection

HeLa cells were cultured at 37 °C and 5% CO_2_ in RPMI-Medium (10% FCS and 1% Penicillin/Streptomycin). 24 h before irradiation, cells were seeded on glass coverslips with an area of (22 × 22) mm^2^ and (170 ± 5) µm thickness (High Precision. Roth. 200,000 cells/well). 2–10 min after post-irradiation incubation at room temperature, cells were fixed by incubation with 2% Paraformaldehyde (PFA) for 15 min. Secondary antibody labeling was performed as described in detail before^23^. In summary, fixed cells were washed with PBS, permeabilized with PBS + Triton (0.15%), and blocked with PBS+ (0.15% Glycine. 1% BSA). For immunostaining, the primary antibodies mouse α-pDNA-PKcs (T2609) (1:350, final concentration 1.43 µg/ml, BioLegend, Catalog # 612902, RRID AB_2315784) and rabbit α-53BP1 (1:350, final concentration 2.9 µg/ml, Novus, Catalog #NB100-305) were incubated at 4 °C overnight. As secondary antibodies, OregonGreen goat α-rabbit (1:400, final concentration 5 µg/ml, Thermo Fisher Scientific, Catalog # O-11038) and Abberior STAR 635P (1:400, final concentration 2.5 µm/ml Abberior, Catalog # ST635-1001-500UG) were incubated at room temperature for 2 h under light-exclusion. The pDNA-PKcs antibody was proven for specifity and quality in immunocytochemistry before^29^. After five times of careful washing with PBS, the coverslips were sealed on glass object slides with Prolong Gold mounting medium and stored at 4 °C until imaging.

### Gamma-irradiation

As reference samples, glass coverslips were placed cells up in the GammaCell ^60^Co Source located at the Helmholtz Center Munich on a specially constructed sample holder. Irradiation was performed with (1.93 ± 0.16) Gy and (3.03 ± 0.25) Gy and lasted only several seconds, preventing the drying of the sample. Due to the high dose rate (> 10 Gy/min) and technical timing limitations, it was not possible to perform defined homogeneous irradiations with lower doses. Cells were placed back into the medium after irradiation and were incubated at room temperature for 3 min, 5 min, 10 min, or 30 min until fixation. The dosimetry was performed with EBT3 Gafchromic films for each sample by placing the film 1 mm above the sample. 48 h after irradiation, the films were scanned using a transmitted light scanner and the darkening in the red channel was analyzed using ImageJ. As a reference curve, proton irradiation of defined doses performed at the ion microprobe SNAKE at the tandem accelerator in Garching was used. It has been shown before that the darkening of the EBT3 is the same for gamma and proton radiation^44^.

### Particle irradiation

Particle irradiation was performed at the ion microprobe SNAKE with the low angle irradiation setup described before^16,36^. Coverslips were dried for 1.5 min at room temperature to ensure a medium layer of max. 10 µm, which is necessary to ensure that the ions reach the cells. After drying, the samples were placed cells upon the sample holder and irradiated under 9° from the top. In total, an area of 4 mm × 22 mm was irradiated. Irradiation was performed with 55 MeV carbon ions, which at the cell surface—due to energy losses in the medium—have an energy of (27 ± 8) MeV and thus a LET of (500 ± 80) $\frac{{\text{keV}}}{\upmu{\text{m}}}$. Irradiation lasted 5 s and was performed with a sublethal mean dose of (1.2 ± 0.4) Gy, as confirmed by checking the fluence between two samples. The mean dose can be calculated as $\text{D}= \frac{\text{F}\times \text{LET}}{{\uprho }_{\text{water}}}$ with a fluence F of (0.015 ± 0.002) $\frac{1}{{\upmu}{\text{m}}^{2}}$ and density of water ${\uprho }_{\text{water}}=1000 \frac{\text{kg}}{{\text{m}}^{3}}$. Further ion irradiations were performed with 33 MeV lithium ions with energy at the cell layer of (20 ± 3) MeV, i.e. a LET of (116 ± 10) $\frac{{\text{keV}}}{\upmu{\text{m}}}$, and a fluence F of (0.011 ± 0.002) $\frac{1}{{\upmu}{\text{m}}^{2}}$. Irradiation was therefore performed with a mean dose of (0.2 ± 0.05) Gy and lasted 5 s, as confirmed by count rate measurements after irradiation of each sample. Simultaneous measuring of count rate and therefore dose was not possible, as the ion ranges are not sufficient to reach the detector, which is placed behind the sample. After irradiation, samples were put back into the medium and incubated at room temperature for 3 min, 5 min, or 10 min until fixation.

### Microscopy and image acquisition

Nanoscopic image acquisition was performed with a commercial CW STED microscope (Leica TCS SP8 3X) with adapted microscopic parameters as described before (Reindl 2017). We utilized an excitation wavelength of 488 nm for the OregonGreen fluorescent dye and a wavelength of 635 nm for the Abberior STAR 635P dye at a power of approximately 1 mW. Detection ranges were 505–560 nm and 640–795 nm, respectively. Depletion was performed with a STED laser beam with a wavelength of 592 nm for the OregonGreen and 775 nm for the Abberior 635P at an approximate power of 50–100 mW. A constant temperature of 23 °C of the sample and the microscope stage was maintained to avoid spatiotemporal shifts according to Reindl et al.^36^. Stacks of whole-cell nuclei (20–40 slices) with a distance of 160 nm were acquired with a 100 × oil objective (Leica HCX PL APO 100×/1.4 Oil) and a pixel size of 40 nm. Dye separation of the raw images for cross-talk minimization was performed with a before-measured unmixing matrix using the Application Suite X (Leica Microsystems) manual unmixing tool. For creating the matrix, single color labeled reference samples were imaged and analyzed.

The unmixed images were deconvolved using a commercial deconvolution program (Huygens software, Scientific Volume Imaging B.V.) as described before^16^, which uses a classical maximum likelihood estimation (CMLE) algorithm with a theoretically calculated point spread function based on the imaging parameters. This gives a lateral resolution of 100 nm and an axial resolution of 200 nm. The resolution was estimated by determining the full width at half maximum of the smallest visible spots on the samples, which are well above the noise and not part of the IRIF.

### Size measurement

IRIF size was measured with a 2D Crosscorrelation plugin for ImageJ as described by Reindl et al.^16^. It is based on the approach introduced by Van Steensel et al.^45^ using the Pearson correlation coefficient

$$r= \frac{{\sum }_{i=1}^{n}\left({A}_{i}-{A}_{mean}\right)\times \left({B}_{i}-{B}_{mean}\right) }{\sqrt{{\sum }_{i=1}^{n}{\left({A}_{i}-{A}_{mean}\right)}^{2}}\times \sqrt{{\sum }_{i=1}^{n}{\left({B}_{i}-{B}_{mean}\right)}^{2}}},$$

with ${A}_{i},{B}_{i}$ the gray values for the i-th pixel in image A and image B, respectively, and ${A}_{mean},{B}_{mean}$ the respective mean gray values. For size measurement, image B is a duplicate of image A, and the cross-correlation function is called the autocorrelation function (ACF). The ACF is obtained by shifting image B over a distance $\Delta x$ over image A and calculating *r* for each pixel shift and plotting $\Delta x$ against *r*. The DNA-PKcs IRIF are round structures, so the fit of a Gaussian function $f\left(x\right)={e}^{-\frac{{x}^{2}}{2\bullet {\sigma }^{2}}}$ to the ACF gives the standard deviation σ as $\frac{1}{2\sqrt{ln2}}$ of the FWHM. The FWHM of the IRIF is obtained as $FWHM=2\sqrt{\text{ln}2}\sigma$ being a factor $\frac{1}{\sqrt{2}}$ smaller than the FWHM of the Gaussian fit to the ACF.

### IRIF counting

The number of IRIF was counted using the Foci Picker 3D Plugin for ImageJ from Du et al.^46^. For each track or cell, the measurement was controlled by the eye, so that only IRIF are counted.

For gamma radiation, the number of DNA-PKcs IRIF and the number of 53BP1 IRIF were measured and compared. In addition, the volume of the cell nucleus was determined by measuring the length, width, and height using ImageJ. This allows for scaling the number of IRIF per cell to IRIF per µm^3^.

For ion irradiation, only the number of DNA-PKcs IRIF was determined, and also the length of the track from first to last 53BP1 signal was measured using ImageJ. With this, the number of IRIF per µm was calculated. For comparison with low-LET control irradiation, the cell nucleus volume was measured and the number of IRIF/µm^3^ was calculated. Here, cells were divided into groups with one, two, or three tracks. This is necessary because the dose to each cell is very different in each case.

### PARTRAC simulation

The present simulations build on the previously published comprehensive database on DNA damage induced by light ions^40,42^. Briefly, model cell nuclei were irradiated by ^1^H, ^4^He, ^7^Li, ^9^Be, ^11^B, ^12^C, ^14^N, ^16^O or ^20^Ne ions with starting energies of 0.25, 0.5, 1, 2, 4, 8, 16, 32, 64, 128, 256 and 512 MeV/u (9 ions × 12 energies = 108 combinations). The ions were started, fully charged, from an 80 µm^2^ circular source tangential to a human lymphocyte nucleus model, a sphere with a 10 µm diameter containing 6.6 Gbp DNA in 23 chromosome pairs. Interactions were scored in a 14.22 µm region of interest, concentric with the nucleus. Interaction cross sections for ions heavier than He were scaled from those for H^47^. Both direct energy depositions and indirect effects via attacks of hydroxyl radicals were considered in the induction of DNA damage, using multi-scale chromatin structures and damage models described previously^38^. In particular, DSB were scored whenever breaks occurred on both strands within 10 bp, and an additional 1% conversion probability of strand breaks to DSB was considered.

Per ion type and energy, tracks were analyzed for at least 1280 particles, up to 40 thousand ions at high energies or 650 thousand high-energy protons, inducing at least 3200 (up to 400 thousand) DSB. To compare with experimentally detected IRIF at early times post-irradiation, the analysis was based on initial geometric coordinates of DSB, with no consideration of their mobility or rejoining during the repair. All DSB induced by a given primary particle (including all its secondary and higher-order electrons) not separated by more than the IRIF size (140 or 600 nm considered here) were assumed to belong to a single cluster. If composed of multiple DSB separated by relatively large distances, the focus might become correspondingly larger and of irregular shape.

In addition to the number of foci, also the (effective) track length within the nucleus was scored. To enable comparison with experimentally detected IRIF, the effective track length was defined here by the separation distance between the farthermost DSB pair induced by the analyzed track. This may differ from the actual track length, which includes also track parts in front of the first and behind the last DSB induced. With increasing LET, however, this difference diminishes. Furthermore for Ions with only one or no DSB a mean track length of 7.6 µm was assumed. This might also differ from the actual track length, but for increasing LET again this error becomes negligible.

## Supplementary Information

Supplementary Information.
