# Supplementary material for: Dosimetry of heavy ion exposure to human cells using nanoscopic imaging of double strand break repair protein clusters
Source: Sci Rep. 2022 Jan 25;12:1305. doi: 10.1038/s41598-022-05413-6 (PMC8789836; doi:10.1038/s41598-022-05413-6)
Supplement: Supplementary file 1 — Supplementary Information. [file 41598_2022_5413_MOESM1_ESM.docx]

Supplementary Material

**Unirradiated control cell**


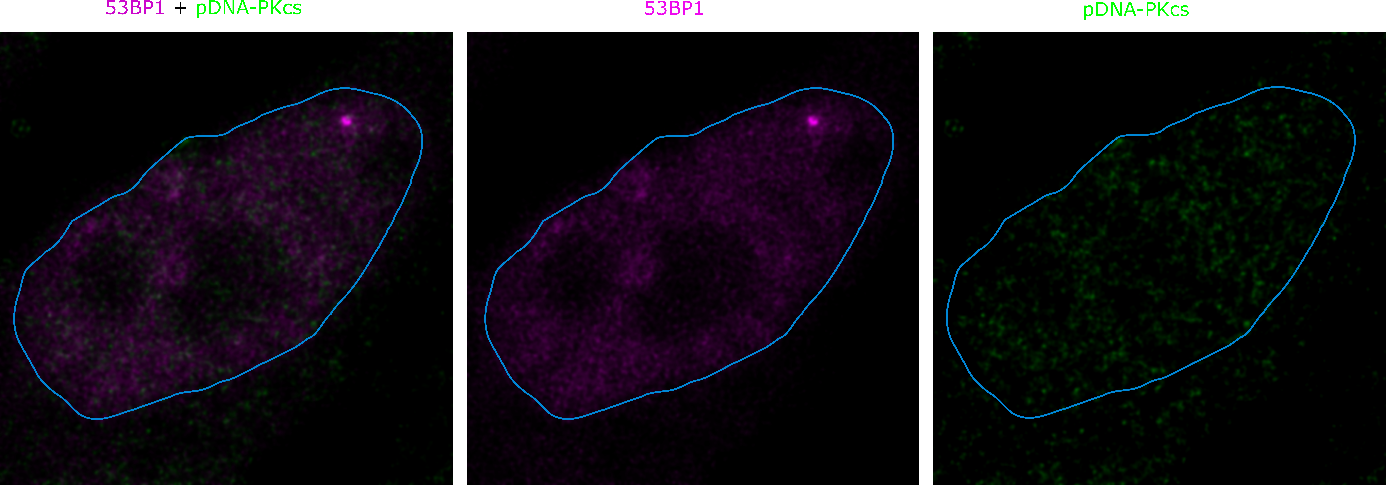


Supplementary Figure 1: 2D plane of a 3D STED image of a non irradiated control cell. 53BP1 is labelled in magenta and pDNA-PKcs in green. A single spontaneous 53BP1 focus is visible. No pDNA-PKcs or double stained foci are visible.

**Box Plots for low-LET irradiation**


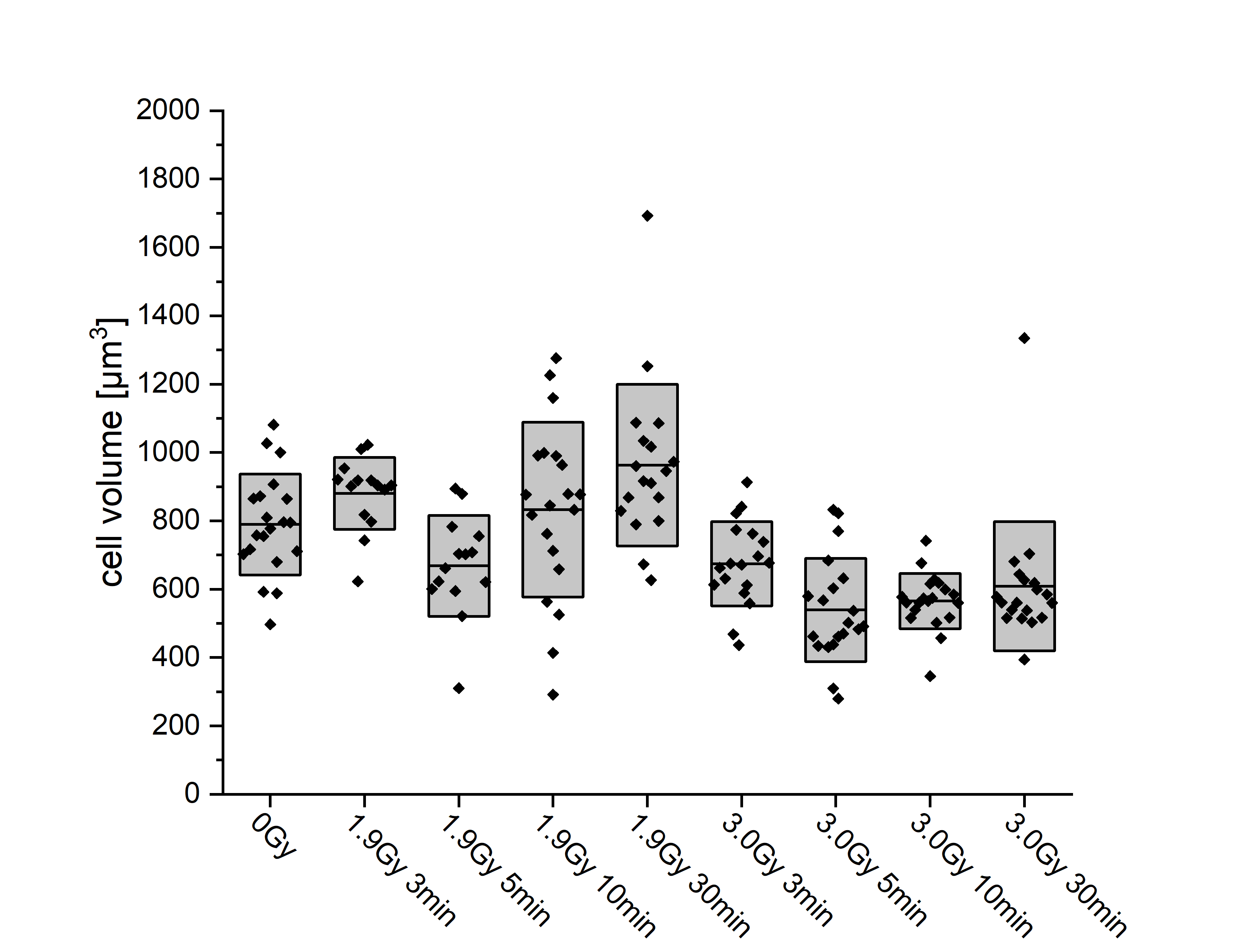

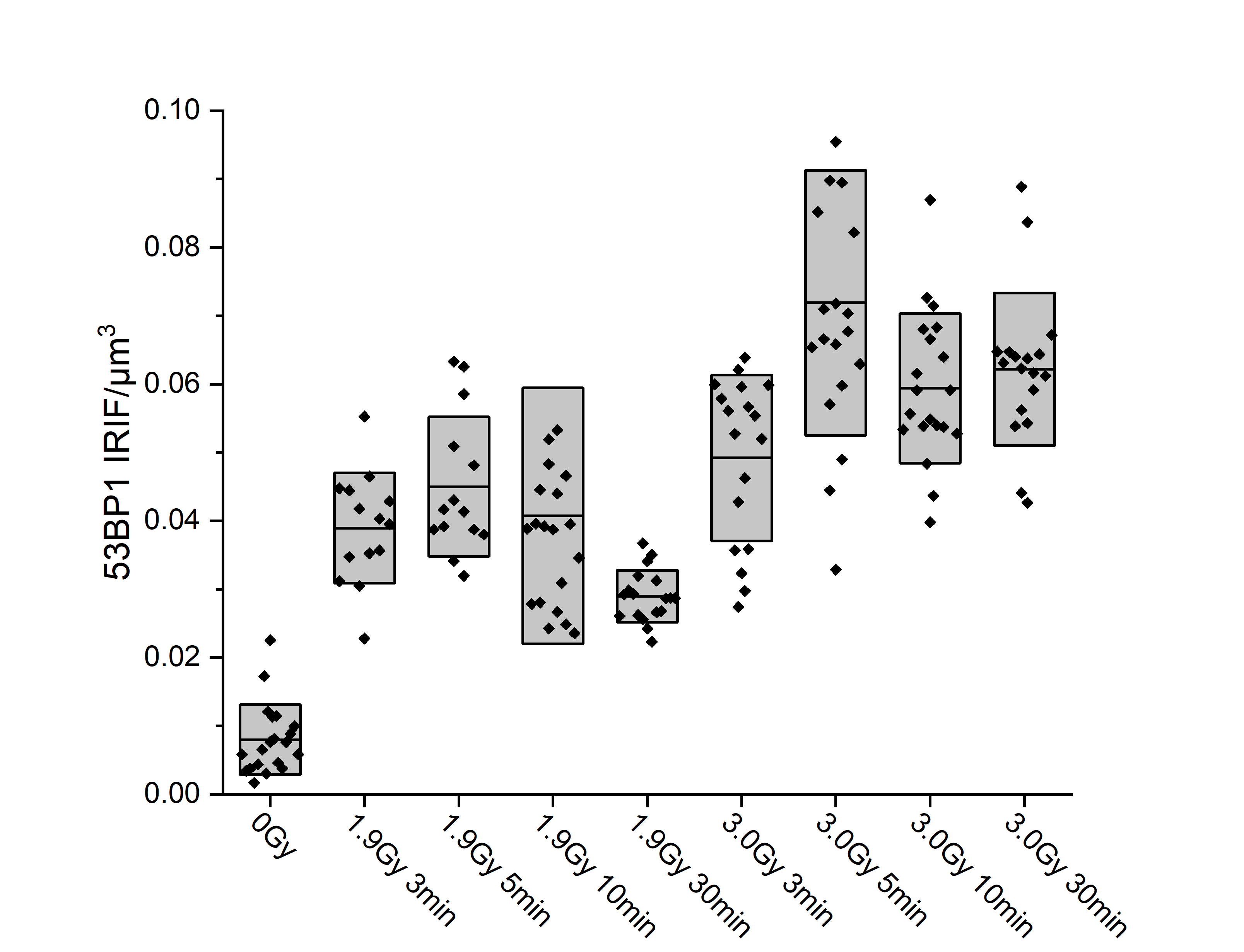

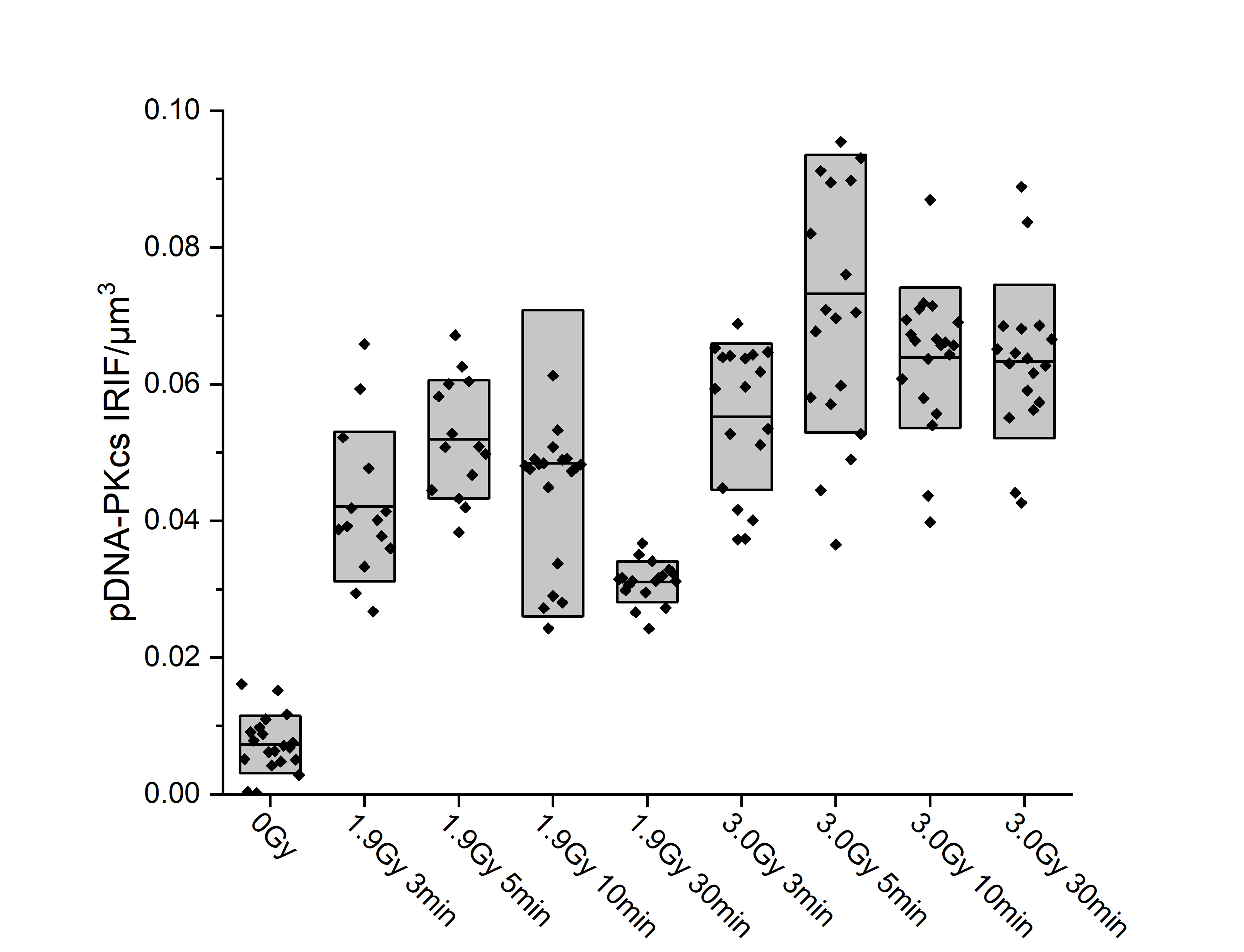


Supplementary Figure 2: Box Plots for the measurements of cell volume and IRIF density for low-LET irradiation. Shown are the raw data used in figure 1c in the manuscript.

**Determining LCD from Simulation**

The location of the first DSB along the simulated ion traversal is used as starting point for the first cluster. As long as a second DSB is within the given finite distance of 140 nm or 600 nm, it is taken within the first cluster. This procedure is then performed with all DSBs until no further DSB is found within the finite distance to one of the DSBs already present in the cluster. Then, the location of the next DSB which is not part of the cluster is searched along the ion track, where the next cluster is started. The procedure is performed until each DSB is part of a cluster.


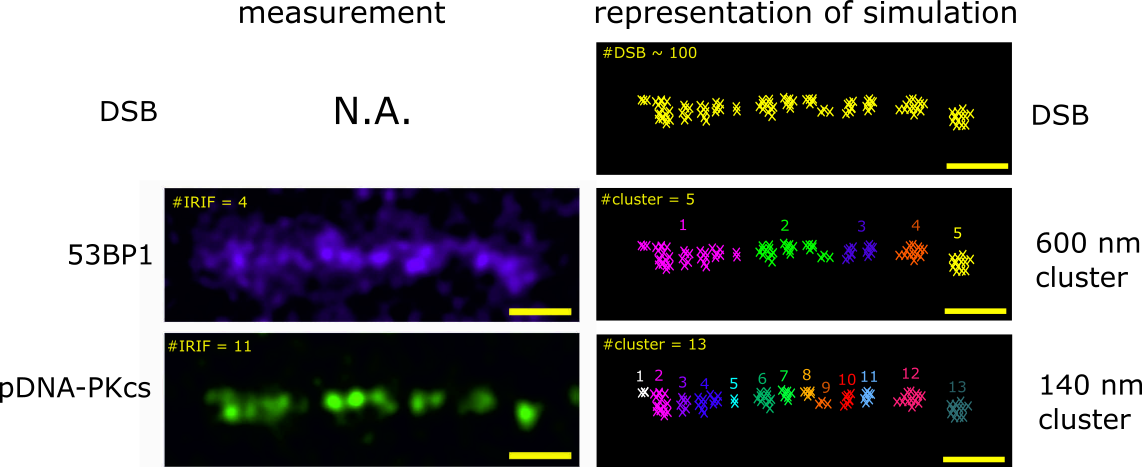


Supplementary Figure 3: Representation of the method of creating LCD from simulation. Left shows a measured carbon ion track from Figure 2. The DSB cannot be measured. 53BP1 (purple) and pDNA-PKcs (green) foci are shown. Measurement gives 4 53BP1 IRIF and 11 pDNA-PKcs IRIF. For the LET of 500 keV/µm simulation gives a value of 20 DSB/µm. Taking the length of the track of approx. 5 µm into account this results in 100 DSB in this track. The predicted 100 DSB within this 5 µm long track are distributed according to the intensity distribution coming from the measurement. The DSB are clustered according to the method described here. This results in 5 600 nm clusters and 13 140 nm clusters. Each cluster is colored differently and labeled with a number for better differentiation. This representation serves as visualization of the performed method.

**High-LET radiation beamtimes**

Supplementary Table 1 Assignments of beamtimes to experiments and total number of analysed cells

| Ion species | Beam time | # cells (for all timepoints) |
| --- | --- | --- |
| Carbon | 05/16 | 20 |
| Carbon | 07/16 | 20 |
| Carbon | 12/16 | 20 |
| Lithium | 07/16 | 20 |
| Lithium | 02/17 | 20 |
| Lithium | 02/17 | 20 |

**Conversion of LCD in LET**

Supplementary Table 2: Table for determination of LET from LCD according to simulation.

| LCD value | | Lower error band | | Upper error band | |
| --- | --- | --- | --- | --- | --- |
| LCD [1/µm] | LET [keV/µm] | LCD [1/µm] | LET [keV/µm] | LCD [1/µm] | LET [keV/µm] |
| 0.00388 | 0.219 | 0.00404 | 0.219 | 0.00373 | 0.219 |
| 0.00394 | 0.22326 | 0.0041 | 0.22326 | 0.00378 | 0.22326 |
| 0.004 | 0.22761 | 0.00416 | 0.22761 | 0.00384 | 0.22761 |
| 0.00406 | 0.23204 | 0.00422 | 0.23204 | 0.0039 | 0.23204 |
| 0.00412 | 0.23656 | 0.00428 | 0.23656 | 0.00396 | 0.23656 |
| 0.00418 | 0.24117 | 0.00435 | 0.24117 | 0.00402 | 0.24117 |
| 0.00425 | 0.24586 | 0.00442 | 0.24586 | 0.00408 | 0.24586 |
| 0.00432 | 0.25065 | 0.00449 | 0.25065 | 0.00414 | 0.25065 |
| 0.00439 | 0.25553 | 0.00456 | 0.25553 | 0.00421 | 0.25553 |
| 0.00446 | 0.2605 | 0.00464 | 0.2605 | 0.00428 | 0.2605 |
| 0.00453 | 0.26558 | 0.00471 | 0.26558 | 0.00435 | 0.26558 |
| 0.00461 | 0.27075 | 0.00479 | 0.27075 | 0.00443 | 0.27075 |
| 0.00469 | 0.27602 | 0.00488 | 0.27602 | 0.0045 | 0.27602 |
| 0.00477 | 0.28139 | 0.00496 | 0.28139 | 0.00458 | 0.28139 |
| 0.00485 | 0.28687 | 0.00505 | 0.28687 | 0.00466 | 0.28687 |
| 0.00494 | 0.29245 | 0.00514 | 0.29245 | 0.00474 | 0.29245 |
| 0.00503 | 0.29815 | 0.00523 | 0.29815 | 0.00483 | 0.29815 |
| 0.00512 | 0.30395 | 0.00532 | 0.30395 | 0.00491 | 0.30395 |
| 0.00521 | 0.30987 | 0.00542 | 0.30987 | 0.005 | 0.30987 |
| 0.00531 | 0.3159 | 0.00552 | 0.3159 | 0.0051 | 0.3159 |
| 0.00541 | 0.32206 | 0.00562 | 0.32206 | 0.00519 | 0.32206 |
| 0.00551 | 0.32833 | 0.00573 | 0.32833 | 0.00529 | 0.32833 |
| 0.00561 | 0.33472 | 0.00584 | 0.33472 | 0.00539 | 0.33472 |
| 0.00572 | 0.34123 | 0.00595 | 0.34123 | 0.00549 | 0.34123 |
| 0.00583 | 0.34788 | 0.00607 | 0.34788 | 0.0056 | 0.34788 |
| 0.00595 | 0.35465 | 0.00619 | 0.35465 | 0.00571 | 0.35465 |
| 0.00607 | 0.36156 | 0.00631 | 0.36156 | 0.00582 | 0.36156 |
| 0.00619 | 0.3686 | 0.00644 | 0.3686 | 0.00594 | 0.3686 |
| 0.00631 | 0.37577 | 0.00657 | 0.37577 | 0.00606 | 0.37577 |
| 0.00644 | 0.38309 | 0.0067 | 0.38309 | 0.00618 | 0.38309 |
| 0.00657 | 0.39055 | 0.00684 | 0.39055 | 0.00631 | 0.39055 |
| 0.00671 | 0.39815 | 0.00698 | 0.39815 | 0.00644 | 0.39815 |
| 0.00685 | 0.4059 | 0.00712 | 0.4059 | 0.00658 | 0.4059 |
| 0.00699 | 0.41381 | 0.00727 | 0.41381 | 0.00671 | 0.41381 |
| 0.00714 | 0.42186 | 0.00743 | 0.42186 | 0.00686 | 0.42186 |
| 0.0073 | 0.43008 | 0.00759 | 0.43008 | 0.007 | 0.43008 |
| 0.00745 | 0.43845 | 0.00775 | 0.43845 | 0.00715 | 0.43845 |
| 0.00761 | 0.44699 | 0.00792 | 0.44699 | 0.00731 | 0.44699 |
| 0.00778 | 0.45569 | 0.00809 | 0.45569 | 0.00747 | 0.45569 |
| 0.00795 | 0.46456 | 0.00827 | 0.46456 | 0.00763 | 0.46456 |
| 0.00813 | 0.47361 | 0.00845 | 0.47361 | 0.0078 | 0.47361 |
| 0.00831 | 0.48283 | 0.00864 | 0.48283 | 0.00798 | 0.48283 |
| 0.0085 | 0.49223 | 0.00884 | 0.49223 | 0.00816 | 0.49223 |
| 0.00869 | 0.50181 | 0.00904 | 0.50181 | 0.00834 | 0.50181 |
| 0.00889 | 0.51158 | 0.00924 | 0.51158 | 0.00853 | 0.51158 |
| 0.00909 | 0.52154 | 0.00946 | 0.52154 | 0.00873 | 0.52154 |
| 0.0093 | 0.5317 | 0.00967 | 0.5317 | 0.00893 | 0.5317 |
| 0.00952 | 0.54205 | 0.0099 | 0.54205 | 0.00914 | 0.54205 |
| 0.00974 | 0.5526 | 0.01013 | 0.5526 | 0.00935 | 0.5526 |
| 0.00997 | 0.56336 | 0.01037 | 0.56336 | 0.00957 | 0.56336 |
| 0.0102 | 0.57433 | 0.01061 | 0.57433 | 0.0098 | 0.57433 |
| 0.01045 | 0.58551 | 0.01086 | 0.58551 | 0.01003 | 0.58551 |
| 0.0107 | 0.59691 | 0.01112 | 0.59691 | 0.01027 | 0.59691 |
| 0.01095 | 0.60853 | 0.01139 | 0.60853 | 0.01052 | 0.60853 |
| 0.01122 | 0.62038 | 0.01167 | 0.62038 | 0.01077 | 0.62038 |
| 0.01149 | 0.63246 | 0.01195 | 0.63246 | 0.01103 | 0.63246 |
| 0.01177 | 0.64477 | 0.01224 | 0.64477 | 0.0113 | 0.64477 |
| 0.01206 | 0.65733 | 0.01254 | 0.65733 | 0.01158 | 0.65733 |
| 0.01236 | 0.67012 | 0.01285 | 0.67012 | 0.01186 | 0.67012 |
| 0.01266 | 0.68317 | 0.01317 | 0.68317 | 0.01215 | 0.68317 |
| 0.01298 | 0.69647 | 0.01349 | 0.69647 | 0.01246 | 0.69647 |
| 0.0133 | 0.71003 | 0.01383 | 0.71003 | 0.01277 | 0.71003 |
| 0.01363 | 0.72386 | 0.01418 | 0.72386 | 0.01309 | 0.72386 |
| 0.01398 | 0.73795 | 0.01454 | 0.73795 | 0.01342 | 0.73795 |
| 0.01433 | 0.75232 | 0.0149 | 0.75232 | 0.01376 | 0.75232 |
| 0.01469 | 0.76696 | 0.01528 | 0.76696 | 0.01411 | 0.76696 |
| 0.01507 | 0.7819 | 0.01567 | 0.7819 | 0.01447 | 0.7819 |
| 0.01545 | 0.79712 | 0.01607 | 0.79712 | 0.01484 | 0.79712 |
| 0.01585 | 0.81264 | 0.01648 | 0.81264 | 0.01522 | 0.81264 |
| 0.01626 | 0.82846 | 0.01691 | 0.82846 | 0.01561 | 0.82846 |
| 0.01668 | 0.84459 | 0.01735 | 0.84459 | 0.01601 | 0.84459 |
| 0.01711 | 0.86104 | 0.0178 | 0.86104 | 0.01643 | 0.86104 |
| 0.01756 | 0.8778 | 0.01826 | 0.8778 | 0.01686 | 0.8778 |
| 0.01802 | 0.89489 | 0.01874 | 0.89489 | 0.0173 | 0.89489 |
| 0.01849 | 0.91231 | 0.01923 | 0.91231 | 0.01775 | 0.91231 |
| 0.01898 | 0.93008 | 0.01973 | 0.93008 | 0.01822 | 0.93008 |
| 0.01948 | 0.94818 | 0.02025 | 0.94818 | 0.0187 | 0.94818 |
| 0.01999 | 0.96664 | 0.02079 | 0.96664 | 0.01919 | 0.96664 |
| 0.02052 | 0.98546 | 0.02134 | 0.98546 | 0.0197 | 0.98546 |
| 0.02107 | 1.00465 | 0.02191 | 1.00465 | 0.02022 | 1.00465 |
| 0.02163 | 1.02421 | 0.02249 | 1.02421 | 0.02076 | 1.02421 |
| 0.02221 | 1.04415 | 0.0231 | 1.04415 | 0.02132 | 1.04415 |
| 0.0228 | 1.06448 | 0.02372 | 1.06448 | 0.02189 | 1.06448 |
| 0.02342 | 1.08521 | 0.02435 | 1.08521 | 0.02248 | 1.08521 |
| 0.02405 | 1.10634 | 0.02501 | 1.10634 | 0.02308 | 1.10634 |
| 0.0247 | 1.12788 | 0.02568 | 1.12788 | 0.02371 | 1.12788 |
| 0.02536 | 1.14984 | 0.02638 | 1.14984 | 0.02435 | 1.14984 |
| 0.02605 | 1.17222 | 0.02709 | 1.17222 | 0.02501 | 1.17222 |
| 0.02676 | 1.19505 | 0.02783 | 1.19505 | 0.02569 | 1.19505 |
| 0.02749 | 1.21831 | 0.02859 | 1.21831 | 0.02639 | 1.21831 |
| 0.02824 | 1.24203 | 0.02937 | 1.24203 | 0.02711 | 1.24203 |
| 0.02901 | 1.26621 | 0.03017 | 1.26621 | 0.02785 | 1.26621 |
| 0.0298 | 1.29087 | 0.03099 | 1.29087 | 0.02861 | 1.29087 |
| 0.03062 | 1.316 | 0.03184 | 1.316 | 0.02939 | 1.316 |
| 0.03146 | 1.34162 | 0.03272 | 1.34162 | 0.0302 | 1.34162 |
| 0.03232 | 1.36774 | 0.03362 | 1.36774 | 0.03103 | 1.36774 |
| 0.03321 | 1.39437 | 0.03454 | 1.39437 | 0.03188 | 1.39437 |
| 0.03413 | 1.42152 | 0.03549 | 1.42152 | 0.03276 | 1.42152 |
| 0.03507 | 1.4492 | 0.03647 | 1.4492 | 0.03367 | 1.4492 |
| 0.03604 | 1.47741 | 0.03748 | 1.47741 | 0.0346 | 1.47741 |
| 0.03703 | 1.50618 | 0.03851 | 1.50618 | 0.03555 | 1.50618 |
| 0.03806 | 1.5355 | 0.03958 | 1.5355 | 0.03654 | 1.5355 |
| 0.03911 | 1.5654 | 0.04068 | 1.5654 | 0.03755 | 1.5654 |
| 0.0402 | 1.59588 | 0.0418 | 1.59588 | 0.03859 | 1.59588 |
| 0.04131 | 1.62695 | 0.04296 | 1.62695 | 0.03966 | 1.62695 |
| 0.04246 | 1.65862 | 0.04416 | 1.65862 | 0.04076 | 1.65862 |
| 0.04364 | 1.69092 | 0.04538 | 1.69092 | 0.04189 | 1.69092 |
| 0.04485 | 1.72384 | 0.04664 | 1.72384 | 0.04306 | 1.72384 |
| 0.0461 | 1.7574 | 0.04794 | 1.7574 | 0.04425 | 1.7574 |
| 0.04738 | 1.79162 | 0.04927 | 1.79162 | 0.04548 | 1.79162 |
| 0.0487 | 1.8265 | 0.05064 | 1.8265 | 0.04675 | 1.8265 |
| 0.05005 | 1.86206 | 0.05205 | 1.86206 | 0.04805 | 1.86206 |
| 0.05144 | 1.89831 | 0.0535 | 1.89831 | 0.04939 | 1.89831 |
| 0.05287 | 1.93527 | 0.05499 | 1.93527 | 0.05076 | 1.93527 |
| 0.05435 | 1.97295 | 0.05652 | 1.97295 | 0.05217 | 1.97295 |
| 0.05586 | 2.01137 | 0.05809 | 2.01137 | 0.05362 | 2.01137 |
| 0.05741 | 2.05053 | 0.05971 | 2.05053 | 0.05511 | 2.05053 |
| 0.05901 | 2.09045 | 0.06137 | 2.09045 | 0.05665 | 2.09045 |
| 0.06065 | 2.13115 | 0.06307 | 2.13115 | 0.05822 | 2.13115 |
| 0.06233 | 2.17264 | 0.06482 | 2.17264 | 0.05984 | 2.17264 |
| 0.06406 | 2.21494 | 0.06662 | 2.21494 | 0.0615 | 2.21494 |
| 0.06584 | 2.25807 | 0.06847 | 2.25807 | 0.06321 | 2.25807 |
| 0.06767 | 2.30203 | 0.07037 | 2.30203 | 0.06496 | 2.30203 |
| 0.06954 | 2.34685 | 0.07232 | 2.34685 | 0.06676 | 2.34685 |
| 0.07147 | 2.39254 | 0.07433 | 2.39254 | 0.06861 | 2.39254 |
| 0.07344 | 2.43913 | 0.07638 | 2.43913 | 0.07051 | 2.43913 |
| 0.07547 | 2.48662 | 0.07849 | 2.48662 | 0.07246 | 2.48662 |
| 0.07756 | 2.53503 | 0.08066 | 2.53503 | 0.07446 | 2.53503 |
| 0.0797 | 2.58439 | 0.08289 | 2.58439 | 0.07651 | 2.58439 |
| 0.08189 | 2.6347 | 0.08517 | 2.6347 | 0.07862 | 2.6347 |
| 0.08415 | 2.686 | 0.08751 | 2.686 | 0.08078 | 2.686 |
| 0.08646 | 2.73829 | 0.08992 | 2.73829 | 0.083 | 2.73829 |
| 0.08883 | 2.79161 | 0.09238 | 2.79161 | 0.08528 | 2.79161 |
| 0.09126 | 2.84596 | 0.09491 | 2.84596 | 0.08761 | 2.84596 |
| 0.09376 | 2.90137 | 0.09751 | 2.90137 | 0.09001 | 2.90137 |
| 0.09632 | 2.95786 | 0.10017 | 2.95786 | 0.09247 | 2.95786 |
| 0.09894 | 3.01545 | 0.1029 | 3.01545 | 0.09499 | 3.01545 |
| 0.10164 | 3.07416 | 0.1057 | 3.07416 | 0.09757 | 3.07416 |
| 0.10439 | 3.13401 | 0.10857 | 3.13401 | 0.10022 | 3.13401 |
| 0.10722 | 3.19503 | 0.11151 | 3.19503 | 0.10293 | 3.19503 |
| 0.11012 | 3.25724 | 0.11453 | 3.25724 | 0.10572 | 3.25724 |
| 0.11309 | 3.32065 | 0.11762 | 3.32065 | 0.10857 | 3.32065 |
| 0.11614 | 3.3853 | 0.12078 | 3.3853 | 0.11149 | 3.3853 |
| 0.11926 | 3.45122 | 0.12403 | 3.45122 | 0.11449 | 3.45122 |
| 0.12245 | 3.51841 | 0.12735 | 3.51841 | 0.11756 | 3.51841 |
| 0.12573 | 3.58691 | 0.13076 | 3.58691 | 0.1207 | 3.58691 |
| 0.12908 | 3.65675 | 0.13424 | 3.65675 | 0.12392 | 3.65675 |
| 0.13251 | 3.72794 | 0.13781 | 3.72794 | 0.12721 | 3.72794 |
| 0.13602 | 3.80053 | 0.14146 | 3.80053 | 0.13058 | 3.80053 |
| 0.13962 | 3.87452 | 0.1452 | 3.87452 | 0.13403 | 3.87452 |
| 0.1433 | 3.94996 | 0.14903 | 3.94996 | 0.13757 | 3.94996 |
| 0.14707 | 4.02686 | 0.15295 | 4.02686 | 0.14118 | 4.02686 |
| 0.15092 | 4.10526 | 0.15696 | 4.10526 | 0.14488 | 4.10526 |
| 0.15486 | 4.18519 | 0.16105 | 4.18519 | 0.14867 | 4.18519 |
| 0.15889 | 4.26668 | 0.16525 | 4.26668 | 0.15253 | 4.26668 |
| 0.16301 | 4.34975 | 0.16953 | 4.34975 | 0.15649 | 4.34975 |
| 0.16723 | 4.43443 | 0.17391 | 4.43443 | 0.16054 | 4.43443 |
| 0.17153 | 4.52077 | 0.17839 | 4.52077 | 0.16467 | 4.52077 |
| 0.17593 | 4.60879 | 0.18297 | 4.60879 | 0.1689 | 4.60879 |
| 0.18043 | 4.69852 | 0.18765 | 4.69852 | 0.17321 | 4.69852 |
| 0.18503 | 4.79 | 0.19243 | 4.79 | 0.17763 | 4.79 |
| 0.18972 | 4.88326 | 0.19731 | 4.88326 | 0.18213 | 4.88326 |
| 0.19451 | 4.97834 | 0.20229 | 4.97834 | 0.18673 | 4.97834 |
| 0.19941 | 5.07526 | 0.20738 | 5.07526 | 0.19143 | 5.07526 |
| 0.2044 | 5.17408 | 0.21258 | 5.17408 | 0.19623 | 5.17408 |
| 0.2095 | 5.27481 | 0.21788 | 5.27481 | 0.20112 | 5.27481 |
| 0.2147 | 5.37751 | 0.22329 | 5.37751 | 0.20611 | 5.37751 |
| 0.22001 | 5.48221 | 0.22881 | 5.48221 | 0.21121 | 5.48221 |
| 0.22542 | 5.58895 | 0.23444 | 5.58895 | 0.2164 | 5.58895 |
| 0.23094 | 5.69776 | 0.24017 | 5.69776 | 0.2217 | 5.69776 |
| 0.23656 | 5.8087 | 0.24603 | 5.8087 | 0.2271 | 5.8087 |
| 0.2423 | 5.92179 | 0.25199 | 5.92179 | 0.23261 | 5.92179 |
| 0.24814 | 6.03709 | 0.25807 | 6.03709 | 0.23821 | 6.03709 |
| 0.25409 | 6.15463 | 0.26426 | 6.15463 | 0.24393 | 6.15463 |
| 0.26016 | 6.27446 | 0.27056 | 6.27446 | 0.24975 | 6.27446 |
| 0.26633 | 6.39662 | 0.27698 | 6.39662 | 0.25568 | 6.39662 |
| 0.27261 | 6.52116 | 0.28352 | 6.52116 | 0.26171 | 6.52116 |
| 0.27901 | 6.64812 | 0.29017 | 6.64812 | 0.26785 | 6.64812 |
| 0.28552 | 6.77756 | 0.29694 | 6.77756 | 0.2741 | 6.77756 |
| 0.29214 | 6.90952 | 0.30382 | 6.90952 | 0.28045 | 6.90952 |
| 0.29887 | 7.04404 | 0.31083 | 7.04404 | 0.28692 | 7.04404 |
| 0.30572 | 7.18119 | 0.31795 | 7.18119 | 0.29349 | 7.18119 |
| 0.31267 | 7.321 | 0.32518 | 7.321 | 0.30017 | 7.321 |
| 0.31974 | 7.46354 | 0.33253 | 7.46354 | 0.30695 | 7.46354 |
| 0.32693 | 7.60885 | 0.34 | 7.60885 | 0.31385 | 7.60885 |
| 0.33422 | 7.757 | 0.34759 | 7.757 | 0.32085 | 7.757 |
| 0.34162 | 7.90802 | 0.35529 | 7.90802 | 0.32796 | 7.90802 |
| 0.34914 | 8.06199 | 0.36311 | 8.06199 | 0.33518 | 8.06199 |
| 0.35677 | 8.21895 | 0.37104 | 8.21895 | 0.3425 | 8.21895 |
| 0.3645 | 8.37897 | 0.37908 | 8.37897 | 0.34992 | 8.37897 |
| 0.37235 | 8.54211 | 0.38724 | 8.54211 | 0.35745 | 8.54211 |
| 0.3803 | 8.70842 | 0.39551 | 8.70842 | 0.36509 | 8.70842 |
| 0.38836 | 8.87797 | 0.40389 | 8.87797 | 0.37282 | 8.87797 |
| 0.39652 | 9.05082 | 0.41238 | 9.05082 | 0.38066 | 9.05082 |
| 0.40479 | 9.22704 | 0.42098 | 9.22704 | 0.3886 | 9.22704 |
| 0.41316 | 9.40669 | 0.42969 | 9.40669 | 0.39664 | 9.40669 |
| 0.42164 | 9.58983 | 0.4385 | 9.58983 | 0.40477 | 9.58983 |
| 0.43021 | 9.77655 | 0.44742 | 9.77655 | 0.413 | 9.77655 |
| 0.43888 | 9.96689 | 0.45643 | 9.96689 | 0.42132 | 9.96689 |
| 0.44764 | 10.16094 | 0.46555 | 10.16094 | 0.42974 | 10.16094 |
| 0.4565 | 10.35877 | 0.47476 | 10.35877 | 0.43824 | 10.35877 |
| 0.46546 | 10.56046 | 0.48408 | 10.56046 | 0.44684 | 10.56046 |
| 0.47451 | 10.76607 | 0.49349 | 10.76607 | 0.45552 | 10.76607 |
| 0.48365 | 10.97568 | 0.50299 | 10.97568 | 0.4643 | 10.97568 |
| 0.49288 | 11.18937 | 0.51259 | 11.18937 | 0.47316 | 11.18937 |
| 0.5022 | 11.40722 | 0.52229 | 11.40722 | 0.48211 | 11.40722 |
| 0.51161 | 11.62932 | 0.53208 | 11.62932 | 0.49115 | 11.62932 |
| 0.52112 | 11.85574 | 0.54196 | 11.85574 | 0.50027 | 11.85574 |
| 0.53071 | 12.08657 | 0.55193 | 12.08657 | 0.50948 | 12.08657 |
| 0.54038 | 12.32189 | 0.562 | 12.32189 | 0.51877 | 12.32189 |
| 0.55014 | 12.56179 | 0.57215 | 12.56179 | 0.52814 | 12.56179 |
| 0.55999 | 12.80637 | 0.58239 | 12.80637 | 0.53759 | 12.80637 |
| 0.56992 | 13.0557 | 0.59271 | 13.0557 | 0.54712 | 13.0557 |
| 0.57992 | 13.30989 | 0.60312 | 13.30989 | 0.55673 | 13.30989 |
| 0.59002 | 13.56903 | 0.61362 | 13.56903 | 0.56641 | 13.56903 |
| 0.60018 | 13.83322 | 0.62419 | 13.83322 | 0.57618 | 13.83322 |
| 0.61043 | 14.10255 | 0.63485 | 14.10255 | 0.58601 | 14.10255 |
| 0.62076 | 14.37712 | 0.64559 | 14.37712 | 0.59592 | 14.37712 |
| 0.63115 | 14.65704 | 0.6564 | 14.65704 | 0.60591 | 14.65704 |
| 0.64163 | 14.9424 | 0.66729 | 14.9424 | 0.61596 | 14.9424 |
| 0.65217 | 15.23333 | 0.67826 | 15.23333 | 0.62608 | 15.23333 |
| 0.66278 | 15.52992 | 0.6893 | 15.52992 | 0.63627 | 15.52992 |
| 0.67347 | 15.83228 | 0.70041 | 15.83228 | 0.64653 | 15.83228 |
| 0.68422 | 16.14053 | 0.71159 | 16.14053 | 0.65685 | 16.14053 |
| 0.69504 | 16.45478 | 0.72284 | 16.45478 | 0.66723 | 16.45478 |
| 0.70592 | 16.77515 | 0.73415 | 16.77515 | 0.67768 | 16.77515 |
| 0.71686 | 17.10176 | 0.74554 | 17.10176 | 0.68819 | 17.10176 |
| 0.72787 | 17.43472 | 0.75698 | 17.43472 | 0.69875 | 17.43472 |
| 0.73893 | 17.77417 | 0.76849 | 17.77417 | 0.70937 | 17.77417 |
| 0.75005 | 18.12023 | 0.78005 | 18.12023 | 0.72005 | 18.12023 |
| 0.76123 | 18.47302 | 0.79168 | 18.47302 | 0.73078 | 18.47302 |
| 0.77246 | 18.83269 | 0.80336 | 18.83269 | 0.74156 | 18.83269 |
| 0.78375 | 19.19936 | 0.8151 | 19.19936 | 0.7524 | 19.19936 |
| 0.79508 | 19.57316 | 0.82689 | 19.57316 | 0.76328 | 19.57316 |
| 0.80647 | 19.95424 | 0.83873 | 19.95424 | 0.77421 | 19.95424 |
| 0.8179 | 20.34275 | 0.85062 | 20.34275 | 0.78518 | 20.34275 |
| 0.82938 | 20.73882 | 0.86255 | 20.73882 | 0.7962 | 20.73882 |
| 0.8409 | 21.14259 | 0.87453 | 21.14259 | 0.80726 | 21.14259 |
| 0.85246 | 21.55423 | 0.88656 | 21.55423 | 0.81836 | 21.55423 |
| 0.86406 | 21.97389 | 0.89863 | 21.97389 | 0.8295 | 21.97389 |
| 0.8757 | 22.40171 | 0.91073 | 22.40171 | 0.84068 | 22.40171 |
| 0.88738 | 22.83787 | 0.92288 | 22.83787 | 0.85189 | 22.83787 |
| 0.89909 | 23.28251 | 0.93506 | 23.28251 | 0.86313 | 23.28251 |
| 0.91084 | 23.73582 | 0.94727 | 23.73582 | 0.8744 | 23.73582 |
| 0.92261 | 24.19795 | 0.95951 | 24.19795 | 0.88571 | 24.19795 |
| 0.93441 | 24.66907 | 0.97179 | 24.66907 | 0.89704 | 24.66907 |
| 0.94624 | 25.14937 | 0.98409 | 25.14937 | 0.90839 | 25.14937 |
| 0.9581 | 25.63902 | 0.99642 | 25.63902 | 0.91978 | 25.63902 |
| 0.96998 | 26.13821 | 1.00878 | 26.13821 | 0.93118 | 26.13821 |
| 0.98188 | 26.64711 | 1.02115 | 26.64711 | 0.9426 | 26.64711 |
| 0.9938 | 27.16592 | 1.03355 | 27.16592 | 0.95405 | 27.16592 |
| 1.00574 | 27.69483 | 1.04597 | 27.69483 | 0.96551 | 27.69483 |
| 1.01769 | 28.23404 | 1.0584 | 28.23404 | 0.97698 | 28.23404 |
| 1.02966 | 28.78375 | 1.07084 | 28.78375 | 0.98847 | 28.78375 |
| 1.04164 | 29.34416 | 1.0833 | 29.34416 | 0.99997 | 29.34416 |
| 1.05363 | 29.91548 | 1.09577 | 29.91548 | 1.01148 | 29.91548 |
| 1.06563 | 30.49793 | 1.10825 | 30.49793 | 1.023 | 30.49793 |
| 1.07763 | 31.09172 | 1.12074 | 31.09172 | 1.03453 | 31.09172 |
| 1.08964 | 31.69706 | 1.13323 | 31.69706 | 1.04605 | 31.69706 |
| 1.10165 | 32.31419 | 1.14572 | 32.31419 | 1.05759 | 32.31419 |
| 1.11367 | 32.94334 | 1.15822 | 32.94334 | 1.06912 | 32.94334 |
| 1.12568 | 33.58474 | 1.17071 | 33.58474 | 1.08066 | 33.58474 |
| 1.1377 | 34.23862 | 1.1832 | 34.23862 | 1.09219 | 34.23862 |
| 1.1497 | 34.90524 | 1.19569 | 34.90524 | 1.10372 | 34.90524 |
| 1.16171 | 35.58483 | 1.20817 | 35.58483 | 1.11524 | 35.58483 |
| 1.1737 | 36.27766 | 1.22065 | 36.27766 | 1.12675 | 36.27766 |
| 1.18569 | 36.98397 | 1.23311 | 36.98397 | 1.13826 | 36.98397 |
| 1.19766 | 37.70404 | 1.24557 | 37.70404 | 1.14976 | 37.70404 |
| 1.20962 | 38.43813 | 1.25801 | 38.43813 | 1.16124 | 38.43813 |
| 1.22157 | 39.18651 | 1.27043 | 39.18651 | 1.17271 | 39.18651 |
| 1.2335 | 39.94946 | 1.28284 | 39.94946 | 1.18416 | 39.94946 |
| 1.24541 | 40.72726 | 1.29523 | 40.72726 | 1.1956 | 40.72726 |
| 1.25731 | 41.52021 | 1.3076 | 41.52021 | 1.20701 | 41.52021 |
| 1.26918 | 42.32859 | 1.31994 | 42.32859 | 1.21841 | 42.32859 |
| 1.28102 | 43.15272 | 1.33227 | 43.15272 | 1.22978 | 43.15272 |
| 1.29285 | 43.99289 | 1.34456 | 43.99289 | 1.24113 | 43.99289 |
| 1.30464 | 44.84942 | 1.35683 | 44.84942 | 1.25246 | 44.84942 |
| 1.31641 | 45.72262 | 1.36907 | 45.72262 | 1.26375 | 45.72262 |
| 1.32815 | 46.61283 | 1.38127 | 46.61283 | 1.27502 | 46.61283 |
| 1.33986 | 47.52036 | 1.39345 | 47.52036 | 1.28626 | 47.52036 |
| 1.35153 | 48.44557 | 1.40559 | 48.44557 | 1.29747 | 48.44557 |
| 1.36317 | 49.38879 | 1.4177 | 49.38879 | 1.30864 | 49.38879 |
| 1.37477 | 50.35038 | 1.42976 | 50.35038 | 1.31978 | 50.35038 |
| 1.38634 | 51.33068 | 1.44179 | 51.33068 | 1.33088 | 51.33068 |
| 1.39787 | 52.33008 | 1.45378 | 52.33008 | 1.34195 | 52.33008 |
| 1.40935 | 53.34893 | 1.46573 | 53.34893 | 1.35298 | 53.34893 |
| 1.4208 | 54.38761 | 1.47763 | 54.38761 | 1.36397 | 54.38761 |
| 1.4322 | 55.44652 | 1.48949 | 55.44652 | 1.37491 | 55.44652 |
| 1.44356 | 56.52605 | 1.5013 | 56.52605 | 1.38582 | 56.52605 |
| 1.45487 | 57.6266 | 1.51307 | 57.6266 | 1.39668 | 57.6266 |
| 1.46614 | 58.74857 | 1.52479 | 58.74857 | 1.40749 | 58.74857 |
| 1.47736 | 59.89239 | 1.53645 | 59.89239 | 1.41826 | 59.89239 |
| 1.48853 | 61.05847 | 1.54807 | 61.05847 | 1.42899 | 61.05847 |
| 1.49965 | 62.24726 | 1.55963 | 62.24726 | 1.43966 | 62.24726 |
| 1.51072 | 63.4592 | 1.57114 | 63.4592 | 1.45029 | 63.4592 |
| 1.52173 | 64.69473 | 1.5826 | 64.69473 | 1.46086 | 64.69473 |
| 1.53269 | 65.95432 | 1.594 | 65.95432 | 1.47138 | 65.95432 |
| 1.5436 | 67.23843 | 1.60534 | 67.23843 | 1.48186 | 67.23843 |
| 1.55445 | 68.54754 | 1.61663 | 68.54754 | 1.49227 | 68.54754 |
| 1.56525 | 69.88214 | 1.62786 | 69.88214 | 1.50264 | 69.88214 |
| 1.57598 | 71.24272 | 1.63902 | 71.24272 | 1.51294 | 71.24272 |
| 1.58666 | 72.6298 | 1.65013 | 72.6298 | 1.5232 | 72.6298 |
| 1.59728 | 74.04388 | 1.66118 | 74.04388 | 1.53339 | 74.04388 |
| 1.60784 | 75.48549 | 1.67216 | 75.48549 | 1.54353 | 75.48549 |
| 1.61834 | 76.95517 | 1.68308 | 76.95517 | 1.55361 | 76.95517 |
| 1.62878 | 78.45346 | 1.69393 | 78.45346 | 1.56363 | 78.45346 |
| 1.63916 | 79.98093 | 1.70472 | 79.98093 | 1.57359 | 79.98093 |
| 1.64947 | 81.53813 | 1.71545 | 81.53813 | 1.58349 | 81.53813 |
| 1.65972 | 83.12565 | 1.72611 | 83.12565 | 1.59333 | 83.12565 |
| 1.66991 | 84.74408 | 1.7367 | 84.74408 | 1.60311 | 84.74408 |
| 1.68003 | 86.39403 | 1.74723 | 86.39403 | 1.61283 | 86.39403 |
| 1.69008 | 88.07609 | 1.75769 | 88.07609 | 1.62248 | 88.07609 |
| 1.70007 | 89.79091 | 1.76808 | 89.79091 | 1.63207 | 89.79091 |
| 1.71 | 91.53911 | 1.7784 | 91.53911 | 1.6416 | 91.53911 |
| 1.71986 | 93.32135 | 1.78865 | 93.32135 | 1.65106 | 93.32135 |
| 1.72965 | 95.13829 | 1.79883 | 95.13829 | 1.66046 | 95.13829 |
| 1.73937 | 96.9906 | 1.80895 | 96.9906 | 1.6698 | 96.9906 |
| 1.74903 | 98.87898 | 1.81899 | 98.87898 | 1.67907 | 98.87898 |
| 1.75862 | 100.80412 | 1.82896 | 100.80412 | 1.68827 | 100.80412 |
| 1.76814 | 102.76675 | 1.83886 | 102.76675 | 1.69741 | 102.76675 |
| 1.77759 | 104.76758 | 1.84869 | 104.76758 | 1.70649 | 104.76758 |
| 1.78697 | 106.80738 | 1.85845 | 106.80738 | 1.71549 | 106.80738 |
| 1.79629 | 108.88688 | 1.86814 | 108.88688 | 1.72444 | 108.88688 |
| 1.80554 | 111.00688 | 1.87776 | 111.00688 | 1.73332 | 111.00688 |
| 1.81472 | 113.16815 | 1.8873 | 113.16815 | 1.74213 | 113.16815 |
| 1.82383 | 115.3715 | 1.89678 | 115.3715 | 1.75087 | 115.3715 |
| 1.83287 | 117.61775 | 1.90618 | 117.61775 | 1.75955 | 117.61775 |
| 1.84184 | 119.90773 | 1.91551 | 119.90773 | 1.76817 | 119.90773 |
| 1.85075 | 122.2423 | 1.92478 | 122.2423 | 1.77672 | 122.2423 |
| 1.85958 | 124.62232 | 1.93397 | 124.62232 | 1.7852 | 124.62232 |
| 1.86835 | 127.04868 | 1.94309 | 127.04868 | 1.79362 | 127.04868 |
| 1.87705 | 129.52227 | 1.95213 | 129.52227 | 1.80197 | 129.52227 |
| 1.88569 | 132.04403 | 1.96111 | 132.04403 | 1.81026 | 132.04403 |
| 1.89425 | 134.61489 | 1.97002 | 134.61489 | 1.81848 | 134.61489 |
| 1.90275 | 137.2358 | 1.97886 | 137.2358 | 1.82664 | 137.2358 |
| 1.91118 | 139.90774 | 1.98763 | 139.90774 | 1.83474 | 139.90774 |
| 1.91955 | 142.6317 | 1.99633 | 142.6317 | 1.84277 | 142.6317 |
| 1.92785 | 145.4087 | 2.00496 | 145.4087 | 1.85074 | 145.4087 |
| 1.93608 | 148.23976 | 2.01353 | 148.23976 | 1.85864 | 148.23976 |
| 1.94425 | 151.12595 | 2.02202 | 151.12595 | 1.86648 | 151.12595 |
| 1.95236 | 154.06832 | 2.03045 | 154.06832 | 1.87427 | 154.06832 |
| 1.9604 | 157.06799 | 2.03882 | 157.06799 | 1.88199 | 157.06799 |
| 1.96838 | 160.12605 | 2.04712 | 160.12605 | 1.88964 | 160.12605 |
| 1.97629 | 163.24366 | 2.05535 | 163.24366 | 1.89724 | 163.24366 |
| 1.98414 | 166.42196 | 2.06351 | 166.42196 | 1.90478 | 166.42196 |
| 1.99193 | 169.66215 | 2.0716 | 169.66215 | 1.91225 | 169.66215 |
| 1.99964 | 172.96542 | 2.07963 | 172.96542 | 1.91966 | 172.96542 |
| 2.00729 | 176.333 | 2.08758 | 176.333 | 1.927 | 176.333 |
| 2.01487 | 179.76615 | 2.09546 | 179.76615 | 1.93427 | 179.76615 |
| 2.02237 | 183.26615 | 2.10327 | 183.26615 | 1.94148 | 183.26615 |
| 2.02981 | 186.83428 | 2.111 | 186.83428 | 1.94862 | 186.83428 |
| 2.03717 | 190.47189 | 2.11866 | 190.47189 | 1.95568 | 190.47189 |
| 2.04446 | 194.18032 | 2.12624 | 194.18032 | 1.96268 | 194.18032 |
| 2.05167 | 197.96095 | 2.13374 | 197.96095 | 1.9696 | 197.96095 |
| 2.05881 | 201.81519 | 2.14116 | 201.81519 | 1.97646 | 201.81519 |
| 2.06587 | 205.74447 | 2.1485 | 205.74447 | 1.98323 | 205.74447 |
| 2.07285 | 209.75025 | 2.15577 | 209.75025 | 1.98994 | 209.75025 |
| 2.07975 | 213.83403 | 2.16294 | 213.83403 | 1.99656 | 213.83403 |
| 2.08658 | 217.99731 | 2.17004 | 217.99731 | 2.00311 | 217.99731 |
| 2.09332 | 222.24165 | 2.17705 | 222.24165 | 2.00959 | 222.24165 |
| 2.09998 | 226.56863 | 2.18398 | 226.56863 | 2.01598 | 226.56863 |
| 2.10655 | 230.97985 | 2.19082 | 230.97985 | 2.02229 | 230.97985 |
| 2.11304 | 235.47696 | 2.19757 | 235.47696 | 2.02852 | 235.47696 |
| 2.11945 | 240.06163 | 2.20423 | 240.06163 | 2.03467 | 240.06163 |
| 2.12577 | 244.73555 | 2.2108 | 244.73555 | 2.04074 | 244.73555 |
| 2.132 | 249.50048 | 2.21728 | 249.50048 | 2.04672 | 249.50048 |
| 2.13815 | 254.35818 | 2.22367 | 254.35818 | 2.05262 | 254.35818 |
| 2.1442 | 259.31046 | 2.22997 | 259.31046 | 2.05844 | 259.31046 |
| 2.15017 | 264.35915 | 2.23618 | 264.35915 | 2.06416 | 264.35915 |
| 2.15604 | 269.50614 | 2.24228 | 269.50614 | 2.0698 | 269.50614 |
| 2.16183 | 274.75335 | 2.2483 | 274.75335 | 2.07535 | 274.75335 |
| 2.16751 | 280.10271 | 2.25422 | 280.10271 | 2.08081 | 280.10271 |
| 2.17311 | 285.55623 | 2.26003 | 285.55623 | 2.08619 | 285.55623 |
| 2.17861 | 291.11592 | 2.26576 | 291.11592 | 2.09147 | 291.11592 |
| 2.18402 | 296.78386 | 2.27138 | 296.78386 | 2.09666 | 296.78386 |
| 2.18932 | 302.56215 | 2.2769 | 302.56215 | 2.10175 | 302.56215 |
| 2.19454 | 308.45294 | 2.28232 | 308.45294 | 2.10675 | 308.45294 |
| 2.19965 | 314.45843 | 2.28764 | 314.45843 | 2.11166 | 314.45843 |
| 2.20466 | 320.58084 | 2.29285 | 320.58084 | 2.11648 | 320.58084 |
| 2.20958 | 326.82245 | 2.29796 | 326.82245 | 2.12119 | 326.82245 |
| 2.21439 | 333.18559 | 2.30297 | 333.18559 | 2.12582 | 333.18559 |
| 2.2191 | 339.67261 | 2.30787 | 339.67261 | 2.13034 | 339.67261 |
| 2.22371 | 346.28593 | 2.31266 | 346.28593 | 2.13477 | 346.28593 |
| 2.22822 | 353.02801 | 2.31735 | 353.02801 | 2.13909 | 353.02801 |
| 2.23262 | 359.90136 | 2.32193 | 359.90136 | 2.14332 | 359.90136 |
| 2.23692 | 366.90853 | 2.3264 | 366.90853 | 2.14745 | 366.90853 |
| 2.24112 | 374.05213 | 2.33076 | 374.05213 | 2.15147 | 374.05213 |
| 2.2452 | 381.33481 | 2.33501 | 381.33481 | 2.15539 | 381.33481 |
| 2.24918 | 388.75929 | 2.33915 | 388.75929 | 2.15921 | 388.75929 |
| 2.25305 | 396.32831 | 2.34318 | 396.32831 | 2.16293 | 396.32831 |
| 2.25682 | 404.0447 | 2.34709 | 404.0447 | 2.16654 | 404.0447 |
| 2.26047 | 411.91133 | 2.35089 | 411.91133 | 2.17005 | 411.91133 |
| 2.26401 | 419.93112 | 2.35458 | 419.93112 | 2.17345 | 419.93112 |
| 2.26745 | 428.10705 | 2.35815 | 428.10705 | 2.17675 | 428.10705 |
| 2.27077 | 436.44217 | 2.3616 | 436.44217 | 2.17994 | 436.44217 |
| 2.27398 | 444.93956 | 2.36494 | 444.93956 | 2.18302 | 444.93956 |
| 2.27707 | 453.6024 | 2.36816 | 453.6024 | 2.18599 | 453.6024 |
| 2.28006 | 462.4339 | 2.37126 | 462.4339 | 2.18885 | 462.4339 |
| 2.28293 | 471.43735 | 2.37424 | 471.43735 | 2.19161 | 471.43735 |
| 2.28568 | 480.61609 | 2.37711 | 480.61609 | 2.19425 | 480.61609 |
| 2.28832 | 489.97354 | 2.37985 | 489.97354 | 2.19678 | 489.97354 |
| 2.29084 | 499.51318 | 2.38247 | 499.51318 | 2.19921 | 499.51318 |
| 2.29324 | 509.23855 | 2.38497 | 509.23855 | 2.20151 | 509.23855 |
| 2.29553 | 519.15327 | 2.38735 | 519.15327 | 2.20371 | 519.15327 |
| 2.2977 | 529.26103 | 2.38961 | 529.26103 | 2.20579 | 529.26103 |
| 2.29975 | 539.56558 | 2.39174 | 539.56558 | 2.20776 | 539.56558 |
| 2.30169 | 550.07076 | 2.39376 | 550.07076 | 2.20962 | 550.07076 |
| 2.3035 | 560.78047 | 2.39564 | 560.78047 | 2.21136 | 560.78047 |
| 2.3052 | 571.6987 | 2.3974 | 571.6987 | 2.21299 | 571.6987 |
| 2.30677 | 582.8295 | 2.39904 | 582.8295 | 2.2145 | 582.8295 |
| 2.30822 | 594.17701 | 2.40055 | 594.17701 | 2.21589 | 594.17701 |
| 2.30956 | 605.74546 | 2.40194 | 605.74546 | 2.21717 | 605.74546 |
| 2.31077 | 617.53914 | 2.4032 | 617.53914 | 2.21834 | 617.53914 |
| 2.31186 | 629.56244 | 2.40433 | 629.56244 | 2.21938 | 629.56244 |
| 2.31283 | 641.81983 | 2.40534 | 641.81983 | 2.22031 | 641.81983 |
| 2.31367 | 654.31586 | 2.40622 | 654.31586 | 2.22112 | 654.31586 |
| 2.3144 | 667.0552 | 2.40697 | 667.0552 | 2.22182 | 667.0552 |
| 2.315 | 680.04256 | 2.4076 | 680.04256 | 2.2224 | 680.04256 |
| 2.31547 | 693.28278 | 2.40809 | 693.28278 | 2.22286 | 693.28278 |
| 2.31583 | 706.78079 | 2.40846 | 706.78079 | 2.2232 | 706.78079 |
| 2.31606 | 720.54159 | 2.4087 | 720.54159 | 2.22342 | 720.54159 |
| 2.31617 | 734.57032 | 2.40882 | 734.57032 | 2.22352 | 734.57032 |
| 2.31616 | 748.87218 | 2.4088 | 748.87218 | 2.22351 | 748.87218 |
| 2.31602 | 763.4525 | 2.40866 | 763.4525 | 2.22338 | 763.4525 |
| 2.31576 | 778.31669 | 2.40839 | 778.31669 | 2.22313 | 778.31669 |
| 2.31537 | 793.47028 | 2.40799 | 793.47028 | 2.22276 | 793.47028 |
| 2.31486 | 808.9189 | 2.40746 | 808.9189 | 2.22227 | 808.9189 |
| 2.31423 | 824.66831 | 2.4068 | 824.66831 | 2.22166 | 824.66831 |
| 2.31347 | 840.72435 | 2.40601 | 840.72435 | 2.22093 | 840.72435 |
| 2.31259 | 857.093 | 2.4051 | 857.093 | 2.22009 | 857.093 |
